# Ethnobotany of medicinal plants used by the Yao people in Gongcheng County, Guangxi, China

**DOI:** 10.1186/s13002-022-00544-6

**Published:** 2022-06-21

**Authors:** Zhaocen Lu, Hailing Chen, Chunrui Lin, Gui Ou, Junsheng Li, Weibin Xu

**Affiliations:** 1 Guangxi Key Laboratory of Functional Phytochemicals Research and Utilization, Guangxi Institute of Botany, Guangxi Zhuang Autonomous Region and Chinese Academy of Sciences, Guilin, 541006 China; 2 Guangxi Key Laboratory of Plant Conservation and Restoration Ecology in Karst Terrain, Guangxi Institute of Botany, Guangxi Zhuang Autonomous Region and Chinese Academy of Sciences, Guilin, 541006 China; 3 Gongcheng Traditional Chinese Medicine Hospital, Guilin, 542500 China

**Keywords:** Ethnomedicine, Medicinal plants, Traditional knowledge, Yao ethnic group

## Abstract

**Background:**

Gongcheng Yao Autonomous County (Gongcheng) is typical for the Yao people in northeastern Guangxi, southern China. The Yao people have a long history of using medicinal plants. In this study, we used ethnobotanical methods to collect traditional knowledge regarding herbal medicines in Gongcheng. Our study provides fundamental data for developing and applying local ethnic medicines and their protection.

**Methods:**

Ethnobotanical data were collected from 103 villages in nine townships from 2014 to 2018 in Gongcheng. A total of 352 informants (279 male and 73 female) were interviewed through semi-structured interviews, key informant interviews, and guided field walks. All the informants were local inhabitants aged between 28 and 101 years of age, of which 40 key informants were selected based on the recommendations of knowledgeable elders and local medical institutions. The informant consensus factor (ICF) was used to evaluate the degree and importance of differences in medicinal plant species and calculated the relative frequencies of citation (RFC) for the recorded medicinal plants.

**Results:**

Data from 352 local healers were collected for the study. The Guanyin and Sanjiang townships had the highest distribution of per capita healers (Pch), while the Gongcheng, Lianhua, and Ping'an townships were relatively lower. Of the 352 local healers, more than half were older than 60 years of age and therefore faced the problem of suitable successors and potential loss of traditional medicinal knowledge. There are 12 types of diseases treated by local healers in the study area, and most of the types had a high ICF value. The highest ICF (0.80) was reported for digestive system disease, followed by urinary system disease (0.78) and nervous system disease (0.77). Traumatic injury and orthopedics, digestive system, and rheumatic disease are the most common ailments. The RFC value calculated in 33 medicinal plant species (with an FC of more than 5) ranged from 0.024 to 0.056. The higher RFC values included *Kadsura longipedunculata*, *Schefflera heptaphylla*, *Plantago asiatica*, etc. The most commonly used medicinal method was decoction; plasters, creams, and some form of moxibustion and cupping skills were locally practiced, but only rarely. The local healers used 306 medicinal plant species (116 families and 255 genera). Herbal plants were most commonly used among these, with whole plants and roots being favored.

**Conclusion:**

The Yao people are highly skilled at using medicinal plants to treat various diseases in Gongcheng. Their treatment methods are varied, convenient, and efficient. Due to the impact of urbanization and economic development, knowledge of traditional medicine is under threat, with declining numbers of local healers and a lack of suitable successors. In order to protect and inherit Yao's traditional medicinal knowledge, it is necessary to educate young healers and to protect biodiversity.

## Background

Traditional medicine currently plays an important role in human health and the fight against the disease. It is common for local healers to excel in using local medicinal plants for disease treatment, especially in mountainous regions or areas inhabited by ethnic minorities where transportation is difficult [1–6]. The most significant advantage of local healers is their proximity and the ability to treat diseases in a timeous manner. Furthermore, they are familiar with the patient's situation and living environment, offering effective treatment [7, 8]. Local healers play an important role in protecting traditional knowledge and biodiversity and local people's health, the development of medicines, and their application [9–12]. In recent years, research regarding medicinal plants and their traditional uses has been increasing worldwide [13–18].

The Yao nationality in China has a long history. Following thousands of years of survival and development, this indigenous population has adapted to the natural environment, and they have created their own medicinal knowledge database, which has played a significant role in their livelihood [19–22]; consequently, it has become an important part of the treasure of Traditional Chinese Medicine. However, historically, the traditional Yao medicinal knowledge has been passed down from generation to generation solely through oral communication. Therefore, considerable Yao medical experience has been lost owing to the natural decline of aged Yao healers. Some Yao medical experience has disappeared before being recorded by the scientific community [23, 24].

**Fig. 1:**
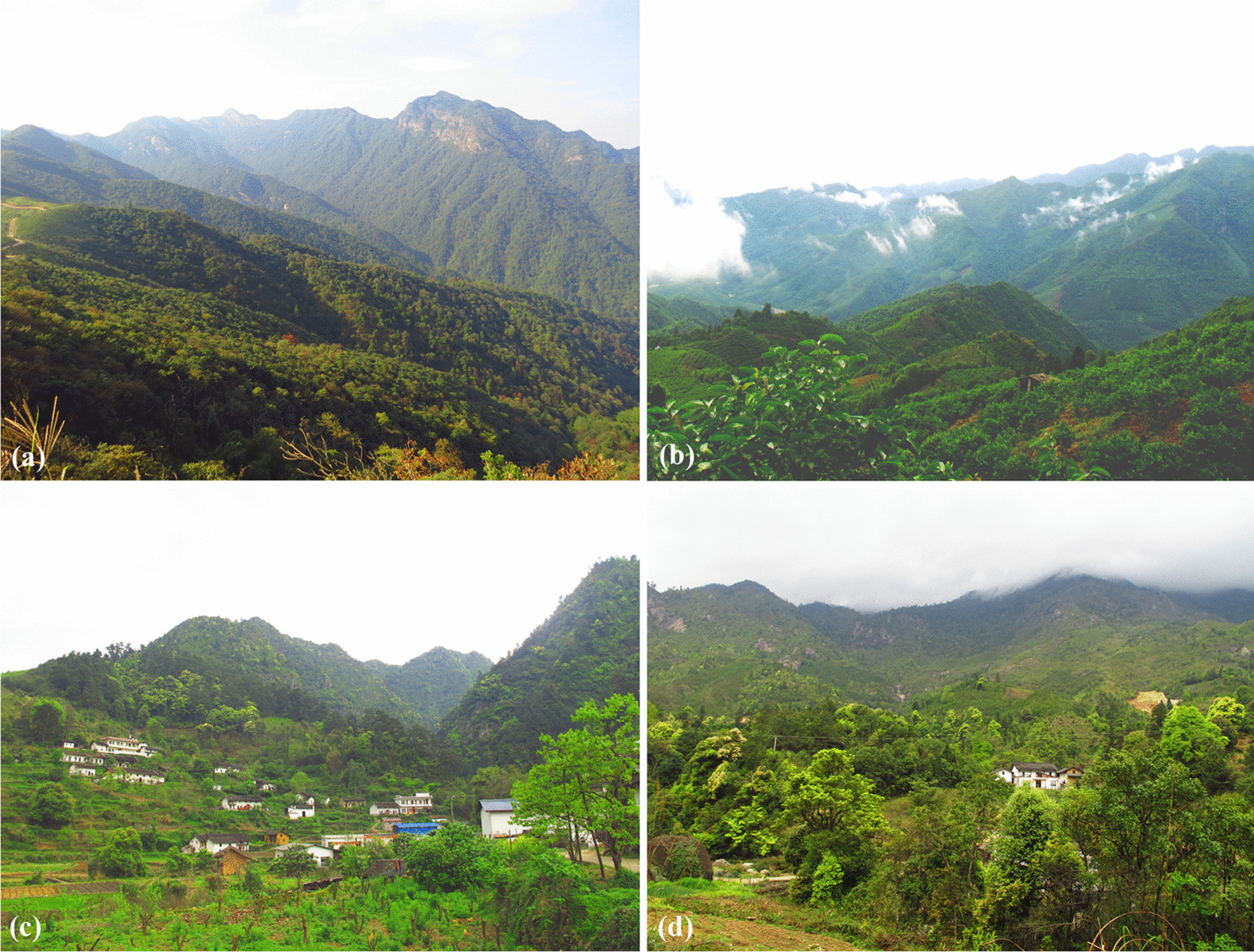
**a** and **b** Yao mountains; **c** and **d** Yao villages and the surrounding farming fields

The Yao people are one of the major ethnic minorities in Guangxi Zhuang Autonomous Region (Guangxi). According to the sixth census, the Yao population in Guangxi has reached 1.49 million, accounting for more than half of the total Yao population in China. Gongcheng Yao Autonomous County (Gongcheng) is an important gathering place for the Yao ethnic group in China and is the second-largest Yao Autonomous County in Guangxi. Here, the Yao population is greater than 148,000, amounting to about 60% of the total population of the county [25], and most parts of the Yao villages are located in the mountains (Fig. 1).

The Yao nationality has a profound cultural heritage and simple folk customs; moreover, many traditional cultural activities have a distinctively local color, for example, the tradition of the "Panwang Festival", "Powang Festival", "Guandi Temple Fair", "Meishan Cultural Festival" and "River Lantern Festival" remains annual customs, and of particular note is the "Herbal medicinal market during the Dragon Boat Festival".

In recent years, during investigations into the herbal medicinal market during the Dragon Boat Festival of Gongcheng, previous authors found that most of the sellers of medicinal materials were middle-aged to older adults, with few young adults [26, 27]. This imbalance is a potential threat to the inheritance of Yao medicine and, therefore, the loss of traditional knowledge. *China Traditional Yao Medicine* and *Yao Ethnic Medicinals in China* [28, 29] are two books published regarding the investigation and study of local Yao medicine in Guangxi. Most of the commonly used prescriptions collected in these two books came from Jinxiu Yao Autonomous County and Du'an Yao Autonomous County in Guangxi. However, these two books rarely included Bama Yao Autonomous County and Fuchuan Yao Autonomous County in Guangxi. The local Yao medicine prescriptions in Gongcheng were also not included in previous investigations.

While advocating the protection of biodiversity and sustainable utilization of resources, attention should also be paid to the protection and inheritance of national traditional knowledge and culture. Nowadays, some traditional knowledge of Yao people from Gongcheng is not documented scientifically and faces disappearing danger. The traditional knowledge regarding herbal medicines in Gongcheng should be preserved as soon as possible. The study aims to grasp the distribution of local healers in Gongcheng and their demographics, analyze the characteristics of the local healers' composition and relate with the traditional knowledge. The current study also used ethnobotanical methods to investigate records of traditional knowledge and experience of ethnic medicine in Gongcheng, obtain first-hand information, record the medicinal plant species used and the types of diseases treated by local healers, analyze the characteristics of species composition and explore the uniqueness of their use methods. This study will provide preliminary data for the development and application of local ethnic medicine and promote the protection inheritance of traditional medicinal knowledge.

## Methods

### Study area and the people

**Fig. 2:**
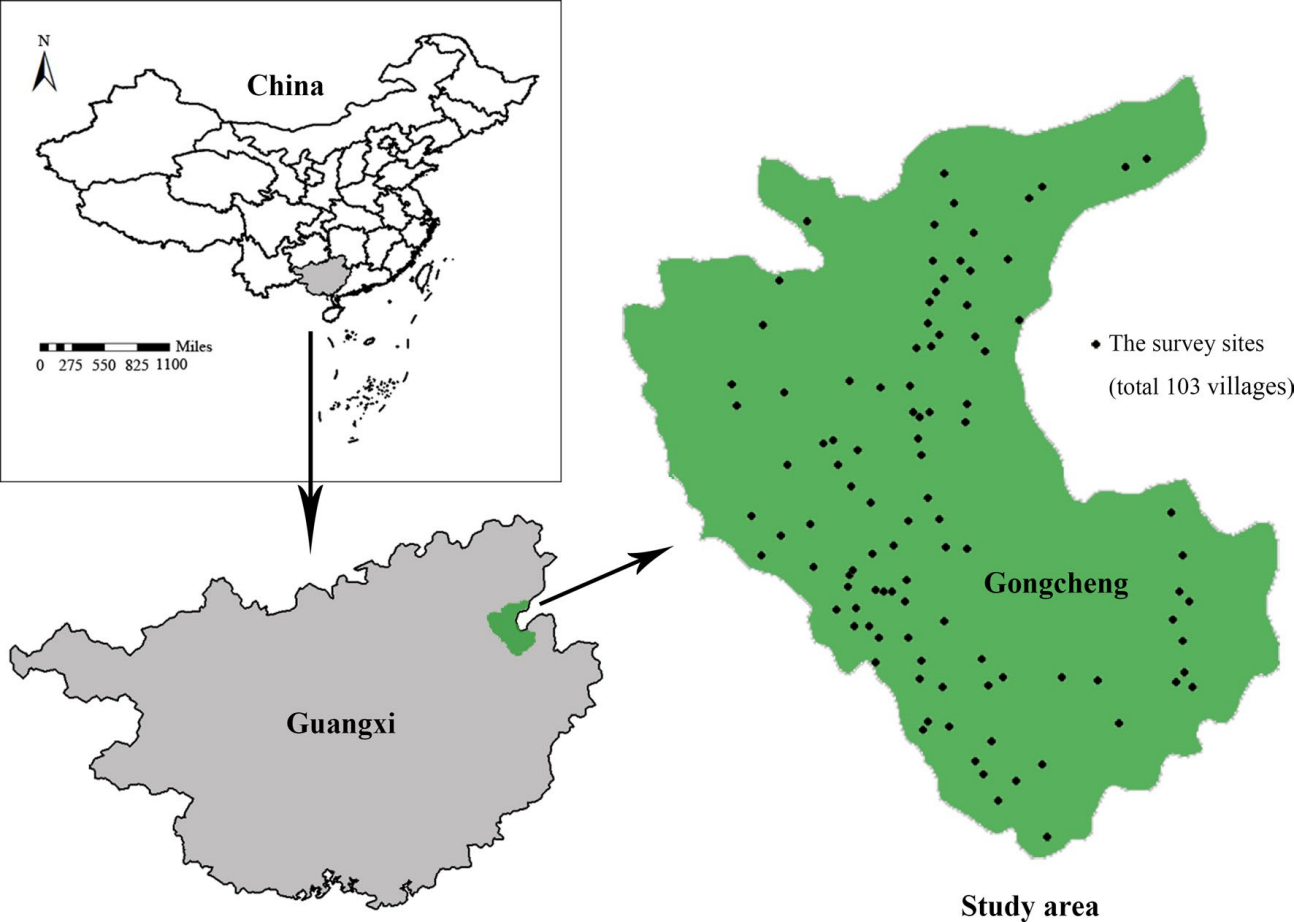
A sketch map of the study area

Gongcheng is located in the northeastern region of Guangxi (Fig. 2). The geographical coordinates are between 24°37′–25°17′ N and 110°36′–111°10′ E. The longest horizontal distance from east to west is 56 km, and the longest longitudinal distance from north to south is 75 km. The county's total area is 2149 km^2^. The administrative level of Gongcheng is county, township, village in descending order and managed by government or committee, so this county has jurisdiction over 117 administrative villages in nine townships [30].

**Fig. 3:**
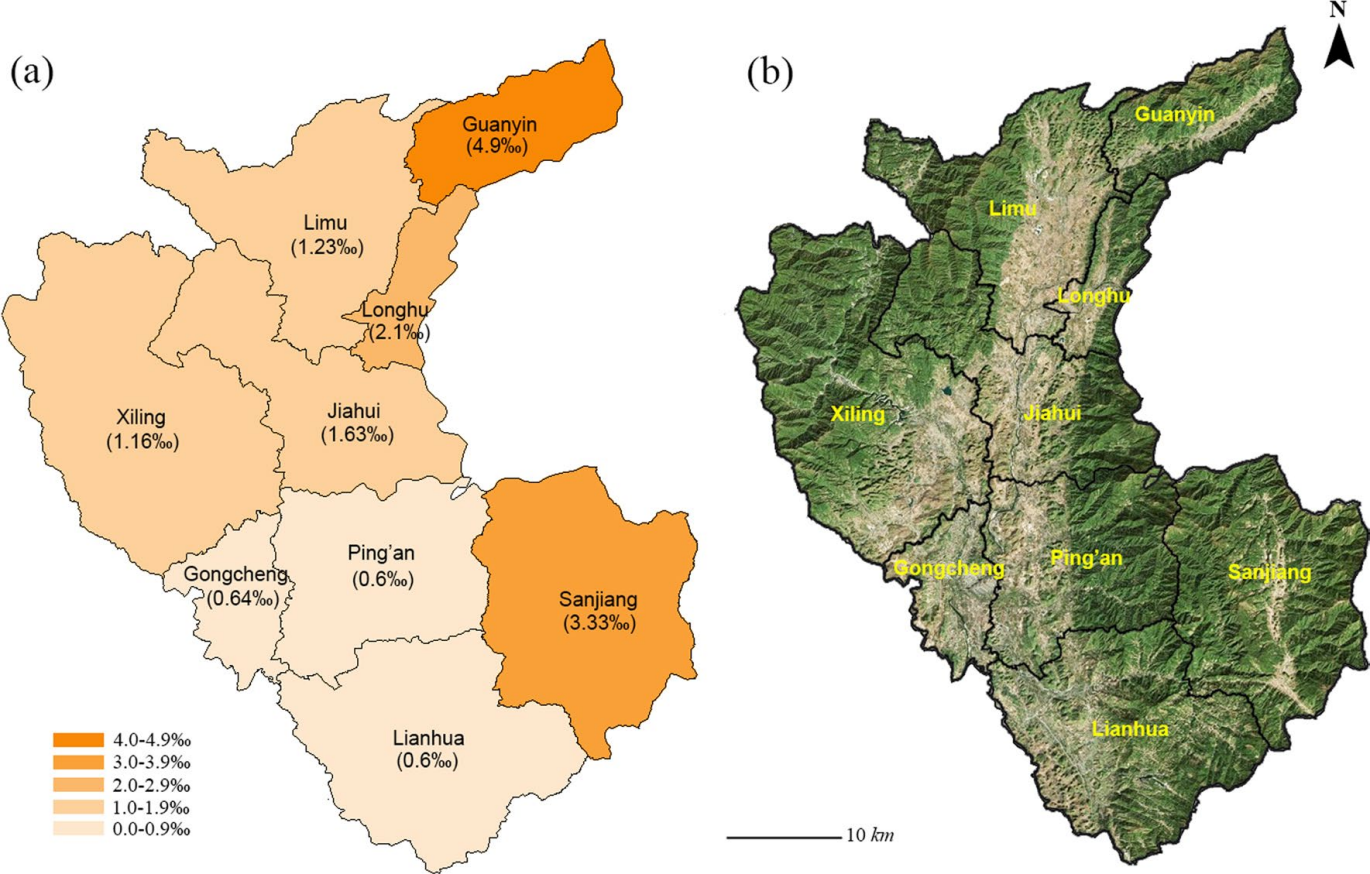
**a** The distribution of "Pch" in the townships of Gongcheng; **b** The topographic map of Gongcheng

The county is located in the Nanling Mountain area, one of southern China’s priority areas for biodiversity conservation. Mengzhu Mountain, Dupang Mountain, and Haiyang Mountain surround the territory, and centrally, there is a huge river corridor scattered with karst landforms of peak clusters and depression (Fig. 3). The vegetation of Gongcheng belongs to the mid-subtropical evergreen forests or mixed evergreen deciduous forests in mountain areas, and bush in karst areas [26, 30]. Influenced by the subtropical monsoon climate, the territory has formed a complex and unique microclimate ecological environment that has nurtured and preserved rich medicinal plant resources that support the Yao people and their medicinal culture.

The local language (Guiliu dialect) is widely used throughout the county and is a common language used by all ethnic groups in Gongcheng. The language family of Yao language varies from place to place and used just in limited areas by older Yao people. The Yao people in Gongcheng has not special or unified religion, just the common worship of nature or ancestral belief, and set some temples, shrines or statues for worship in each village. The Yao people in Gongcheng mainly live on traditional agriculture, e.g., rice, corn. The economic forests are very important source of finance in mountain areas, such as Chinese fir, pine, moso bamboo, and oil-tea camellia. [26, 30].

Due to remote mountainous areas and poor economic condition, those traditional remedies of medicinal plants are the most important therapeutics for the Yao people in Gongcheng. The traditional knowledge of medicinal plants with long utilization history had supported their livelihoods. The local healers in Gongcheng have developed their own ethnomedicinal knowledge and treat patients in their villages or near villages. These local healers were hardly by formal or informal trained, and their medical knowledge is mainly acquired through family inheritance or another healers and self-study. The specialized healers are engaged in treatment work in private clinics or hospitals in township, but they are in the minority. The non-specialized healers are mainly engaged in agriculture and treat patients at sparetime.

### Data collection

After getting agreements from the local government and local healers, a total of 352 informants (279 male and 73 female) were interviewed in the study area, of which 312 were selected using the snowball technique, and 40 key informants were specifically selected based on the recommendations of knowledgeable elders and local medical institutions. The key informants were local famous healers who have rich medical experience, good curative effect and were important custodians and participants of the knowledge of indigenous medicine. All informants were local inhabitants aged between 28 and 101 years. The ethnobotanical investigations were carried out to collect data regarding medicinal plants used to treat human diseases following the methods of the Yao people.

We used semi-structured interviews, key informant interviews, and guided field walks to collect information. The ethnobotanical data were collected from 2014 to 2018. The questionnaire included the name, gender, nationality, age, family address, contact information, and other information of the local healers, as well as the diseases that can be treated effectively. Investigation and interview of key informants included information regarding the diseases, compatibility of medicinal materials, processing and treatment methods, taboos, and means of succession of information. In addition, the key informants were asked to perform preference ranking exercises. We followed the local Yao healers during their collection of herbs in the field and recorded the names, usages, and parts of the medicinal plants used.

### Specimen collection and identification

Field observations were performed with local healers to identify the morphological features and habitats of each medicinal plant species. Voucher specimens and photographs of the local medicinal plants were collected from the field, herbal medicinal market, or home gardens, and the life forms and habitats of these plants were recorded. For future reference, voucher specimens were deposited in the Herbarium of Guangxi Institute of Botany (IBK), Guilin, Guangxi, China.

Voucher specimens and photographs were identified and confirmed according to *Flora of China*, *Flora of Guangxi*, and other botanical websites such as https://www.cvh.ac.cn/, http://www.nsii.org.cn/2017/, http://www.iplant.cn/frps, and https://www.ipni.org/. Finally, specimens that were difficult to identify were discussed with consulting taxonomic experts, and the final inventory of medicinal plants was completed.

### Data analysis

The ethnobotanical data were analyzed and summarized using a Microsoft Office Excel sheet and statistical methods. The key informants shortlisted the plants in this study, and then, their importance in managing diseases was discussed. The preference ranking method was used to rank the diseases, application methods, medicinal parts, and the life forms of the medicinal plants used by the local Yao healers in the study area [31].

The data per capita healers (Pch) of each township were calculated for the data of each township healer (person) divided by the population (thousands) of the township. If the Pch was equal to 1, that indicated one healer for an average of 1000 people. The formula used was

$${\text{Pch}}\;(\permil) = {\text{H}}({\text{p}})/{\text{P}}({\text{t}})$$

The informant consensus factor (ICF) was used to analyze the difference of medicinal plant species used by different healers to treat a particular disease category [32]. The formula is listed below:

$${\text{ICF}} = \left( {{\text{nur}} - {\text{nt}}} \right)/\left( {{\text{nur}} - 1} \right)$$

where nur is the sum of the number of plant species used by all informants to treat a particular disease category, and nt is the total number of plant species commonly used by all informants to treat a particular disease category.

The relative frequency of citation (RFC) was used to evaluate the important plant species used by local healers to treat various diseases. The formula is listed below:

$${\text{RFC}} = {\text{FC}}/{\text{N}}$$

where FC is the number of prescriptions mentioning the use of plant species, and N is the total number of prescriptions in this survey [33].

## Results and discussion

### Distribution of local healers in the study area

Information on a total of 352 local healers was collected through our survey, which was distributed across 103 villages in nine townships in Gongcheng (Fig. 2). According to the statistics at the township level, Limu had the largest population of 54 local healers, followed by Sanjiang (48), Guanyin (47), and Xiling (45). Forty-three local healers were in Jiahui, 35 in Lianhua, 35 in Gongcheng, 23 in Ping'an, and 22 in Longhu (Table 1). The value of per capita healers (Pch) was calculated, and the highest value was noted in Guanyin (4.90‰), followed by Sanjiang (3.33‰), Longhu (2.10‰), Jiahui (1.63‰), Limu (1.23‰), and Xiling (1.16‰), and the lowest values were in Gongcheng, Lianhua, and Ping'an, at around 0.60‰ (Table 1, Fig. 3). At the village level, the Shuibin village in Guanyin township has the most local healers with 24 people, followed by Shitang village in Guanyin township with 16 people and Sanlian village in Sanjiang township with 11 people. There are near 30 villages with just only one or two local healers from Gongcheng, Lianhua, and Ping'an townships.

**Table 1:** The number of local healers in townships within Gongcheng

Township	Area (10 km^2^)	Population (thousand)	Healers (person)	Pch (‰)
Guanyin	14.7	9.6	47	4.90
Sanjiang	29.6	14.4	48	3.33
Longhu	58.0	10.5	22	2.10
Jiahui	24.8	26.3	43	1.63
Limu	27.7	43.9	54	1.23
Xiling	43.1	38.8	45	1.16
Gongcheng	9.0	54.5	35	0.64
Lianhua	36.1	58.2	35	0.60
Ping’an	24.0	38.2	23	0.60

The population data are from the statistics of *Gongcheng annals* in 2012

From the data, we found that the Gongcheng, Lianhua, and Ping'an townships had relatively low Pch, at about 0.60‰. Because the Gongcheng township is the seat of the county government, and the Lianhua and Ping'an townships are close to the Gongcheng township, these three townships have undergone the highest degree of urbanization, modern construction and economic development in recent years and are more influenced by modern Chinese and Western medicine. Hence, the number of local healers in these townships today is lower. The Guanyin and Sanjiang townships now have the highest distribution of Pch, reaching 4.90‰ and 3.33‰, respectively. These two townships are typical minority nationality townships, with the population of Yao nationality accounting for more than 90%. These results indicate that these areas with a denser population of Yao nationality had greater preservation of local healers and medicinal knowledge and must as key areas for the protection inheritance of traditional medicinal knowledge. In addition, the Guanyin township is located in the extreme north of Gongcheng and the Du Pangling Mountains. The Sanjiang township is located in southeastern Gongcheng and south of the Yindian mountains. These two townships are located in remote mountainous areas, are populated with many Yao people, and have a relatively low degree of economic development. With continuing urbanization and economic development, the succession and inheritance of local healers and traditional knowledge have been disregarded in recent years. Therefore, the protection and inheritance of traditional knowledge should be strengthened as quickly as possible, especially in Guanyin and Sanjiang townships.

### Demographics of the informants

**Fig. 4:**
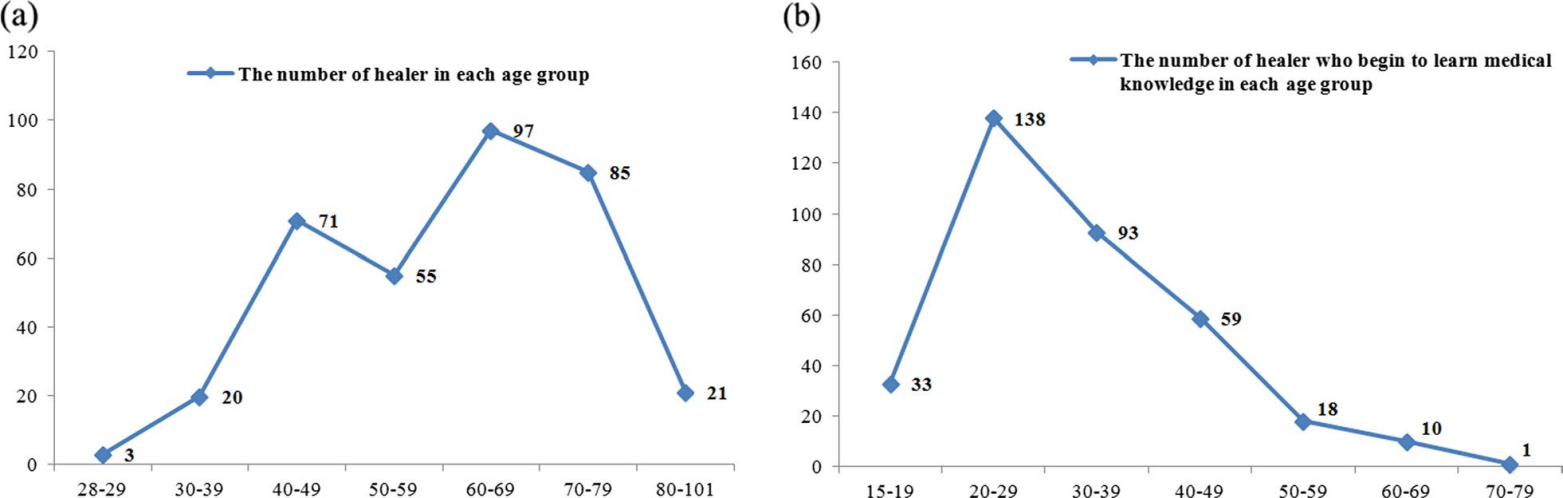
**a** The number of healer in each age group in Gongcheng; **b** The number of healer who begin to learn medical knowledge in each age group in Gongcheng

Among the 352 local healers in the study area, 279 (79.26%) were male, and 73 (20.74%) were female. This is owing to the conservative inheritance of Yao medicinal knowledge, and the custom of passing knowledge on to male members, rather than female members of the society; a matter which is also related to the fact that women are predominantly engaged in housework and agricultural work, while men are mostly engaged in physical and technical labor. Concerning age, the oldest healer was a 101-year-old man from Changjia village in Limu township, who had been a healer for more than 60 years. The youngest was a woman aged 28 years, a healer for nearly 5 years. The ages were mostly between 40 and 79 years (n = 308), while only 23 healers were 28–39 years old, and only 21 were 80–101 years old. Notably, the number of young healers was very low (Fig. 4). Of all healers, those aged 60–69 years were the highest number (27.56%), followed by those aged 70–79 years (24.15%). We counted the number of healer who begin to learn medical knowledge in each age group and found that 20–29 age group was the most (n = 138), followed by 30–39 age group (n = 93) and 40–49 age group (n = 59) (Fig. 4). The number of people in the 20–39 age group is 231 (65.63%), which shows that most healers in Gongcheng begin to learn medical knowledge from the young age. In contrast, the current statistics about healers of all age groups show that young healers are in the minority. Therefore, more than half of the local healers were exceeding 60 years, and the lack of succession and inheritance of Yao medicine is evident. One reason for this phenomenon is that the manner of succession is quite conservative, in that passing on knowledge to external sources and female members are generally restricted. Furthermore, there is no written language of the Yao ethnic group, so the inheritance depends on oral transmission, and unfortunately, the great traditional knowledge has not been passed down by written records. Moreover, young people are resistant to learning traditional knowledge, as they feel it is outdated, useless, and a source of only meager income. They are more willing to travel great distances for work that will give them a higher income or learn Chinese medicine and Western medicine, which are generally more acceptable to the public. This phenomenon also occurs in other areas, with some facing the serious threat of losing their inherited traditions [7, 8, 14, 34, 35].

Most of the famous and old healers in this survey are excellent representatives of local Yao healers in Gongcheng. Over their lifetimes, they continually accumulated practical experience from their therapeutic activities and also absorbed the experience of predecessors. This precious wealth of Yao medicine plays an important role in inheritance, innovation, and development. Furthermore, it is because of the accumulation and inheritance of the experience of Yao medicine from past generations that Yao medicine has developed and remained relevant in modern times. Therefore, these practices should be actively encouraged, and in-depth investigations and excavations should be implemented to avoid the decline in successors and loss of precious traditional experience.

### Diseases treated in the study area

After sorting and statistical analysis, 352 local healers demonstrated a good history of treating diseases, which could be classified into 12 categories based on the eight systems of the human body and the medication characteristics of the Yao people. Gongcheng had the largest number of healers (176, 50%) who were successful in treating traumatic injury and orthopedics, followed by digestive system disease (101, 28.69%), skin and facial disease (93, 26.42%), and rheumatic disease (91, 25.85%) (Table 2).

**Table 2:** The types of diseases treated by local healers

Category	Disease	Number of healers who effectively treated diseases	Percentage of total healers (%)
Traumatic injury and Orthopedics	Hyperostosis (72), Traumatic injury (69), Orthopedics (fracture, lumbocrural pain, muscle and bone pain) (35)	176	50.00
Digestive system disease	Liver disease (hepatitis, liver ascites) (34), Gastropathy (gastritis, gastric ulcer, gastric hemorrhage) (23), Enteritis (diarrhea, dysentery) (17), Cholecystitis (2), Pancreatitis (1), Hemorrhoids (10), Typhoid (14)	101	28.69
Skin and facial disease	Skin diseases (herpes zoster, eczema, scabies, urticaria) (47), Undefined swelling and soreness (26), Burn and scald (12), Toothache (4), Eye disease (3), Earache (1)	93	26.42
Rheumatic disease	Rheumatism (90), Scapulohumeral periarthritis (1)	91	25.85
Gynecological disorders	Gynecological disorders (irregular menstruation, metrorrhagia, infertility) (46), Mastitis (10)	56	15.91
Urinary system disease	Nephritis (8), Stone (47)	55	15.63
Nervous system disease	Snake bite (37), Diseases of acupuncture and massage department (4), Epilepsy (2), Migraine (1)	44	12.50
Pediatric	Pediatric (infantile malnutrition, fever, jaundice, convulsion) (41)	41	11.65
Circulation system disease	Hypertension (5), Heart disease (3), Anemia (1), Stroke (cerebral infarction) (24)	33	9.38
Respiratory system disease	Cold (wind-cold, high fever) (17), Pharyngitis (7), Pneumonia (5), Rhinitis (2)	31	8.81
Immune system disease	Lymphadenitis (8), Rheumatoid arthritis (3), Diabetes (2)	13	3.69
Others	Gray hair (1), Fatigue (1), Male infertility (1), Dog bite (1), Bald spot (1), Alopecia (1)	6	1.70

Traumatic injury and orthopedics were the most common diseases that local healers effectively treated; these were related to local people being engaged in agricultural and forestry production; this type of labor commonly results in mechanical injuries, knife wounds, and fractures. Rheumatism, hyperostosis, traumatic injury, lithiasis, skin diseases, gynecological disorders, pediatrics, snake bites, orthopedics, and liver disease were commonly mentioned in the survey. According to the results, more than 30 local healers effectively treated these common diseases effectively, especially rheumatism and hyperostosis, which were resolved by more than 70 local healers. As these diseases are common, local healers must treat them timely, convenient, and efficiently to improve outcomes.

The public has recognized the unique curative effect of Yao medicine through the historical accumulation of experience with such diseases. Gynecological and pediatric diseases are common in the daily lives of the Yao. The various gynecological drugs commonly used include *Campsis grandiflora*, *Hedyotis caudatifolia*,　*Nuphar pumila*, *Saururus chinensis*, and *Dichroa febrifuga*. Furthermore, pediatric drugs including *Primulina fimbrisepala*, *Ilex rotunda*, *Siphonostegia chinensis*, *Polygala polifolia*, and *Striga asiatica* are potent and convenient for Yao healers who prefer to use fresh herbs as materials.

Disease incidence is often closely related to the local environment and climate, as well as ethnic activities and lifestyles. According to the survey, Gongcheng had the largest number of healers who could effectively treat rheumatic disease because Gongcheng is located in the south of the Nanling Mountains, where the high mountains, dense forests, high temperature, rainy weather, wind, cold, and damp heat are conducive to the development of rheumatism. Moreover, the ancestors of the Yao people frequently migrated to higher elevations with dense forests in the mountains. Life in these mountainous regions is tough, and traumatic injuries, snake bites, and insect bites are frequent occurrences; in addition, skin diseases and orthopedic diseases such as fractures, lumbocrural pain, and muscle or bone pain are locally common. Thus, the local healers' ability to treat such diseases has increased. Similar results have also been found in other minority areas in southern China [7, 8, 36, 37].

### Informant consensus factor

The ICF was calculated for each disease category, ranging from 0.44 to 0.80 (Table 3). The highest ICF (0.80) was reported for digestive system disease with 20 species and 98 use reports, followed by urinary system disease (0.78) with 9 species and 37 use reports, nervous system disease (0.77) with 8 species and 32 use reports, skin and facial disease (0.75) with 19 species and 74 use reports, and pediatric (0.75) with 9 species and 33 use reports, etc.

**Table 3:** Informant consensus factor (ICF) by categories of diseases in the study area

Categories	nur	nt	ICF
Digestive system disease	98	20	0.80
Urinary system disease	37	9	0.78
Nervous system disease	32	8	0.77
Skin and facial disease	74	19	0.75
Pediatric	33	9	0.75
Immune system disease	16	6	0.67
Traumatic injury and Orthopedics	110	42	0.62
Gynecological disorders	62	27	0.57
Respiratory system disease	51	23	0.56
Circulatory diseases	19	11	0.44
Rheumatic disease	67	38	0.44

The higher the ICF value, the higher the diversity of plant species used by healers to treat a particular disease category. The lower the ICF value, the more concentrated the plant species used by healers to treat a particular disease category [32]. Most disease categories had a high ICF value (near 1). The digestive system disease had the highest ICF value; this was probably related to the local healers obtaining a diversity of medicinal plants from wild habitats, while having little communication with other healers, during the conservative inheritance of medicinal knowledge. There were 110 plant species used to treat traumatic injury and orthopedic diseases, and this was likely related to the local people being prone to traumatic injury, hyperostosis, knife wound and fracture when engaged in agricultural and forestry production. Therefore, the healers were required to use a variety of plants for treatment when dealing with these emergencies. The lowest ICF was for circulatory and rheumatic disease, which was probably related to the long treatment cycle of these diseases. During long-term treatments, local healers had a high consensus on the species of medicinal plants used.

### Methods of treatment and ethnic characteristics

**Fig. 5:**
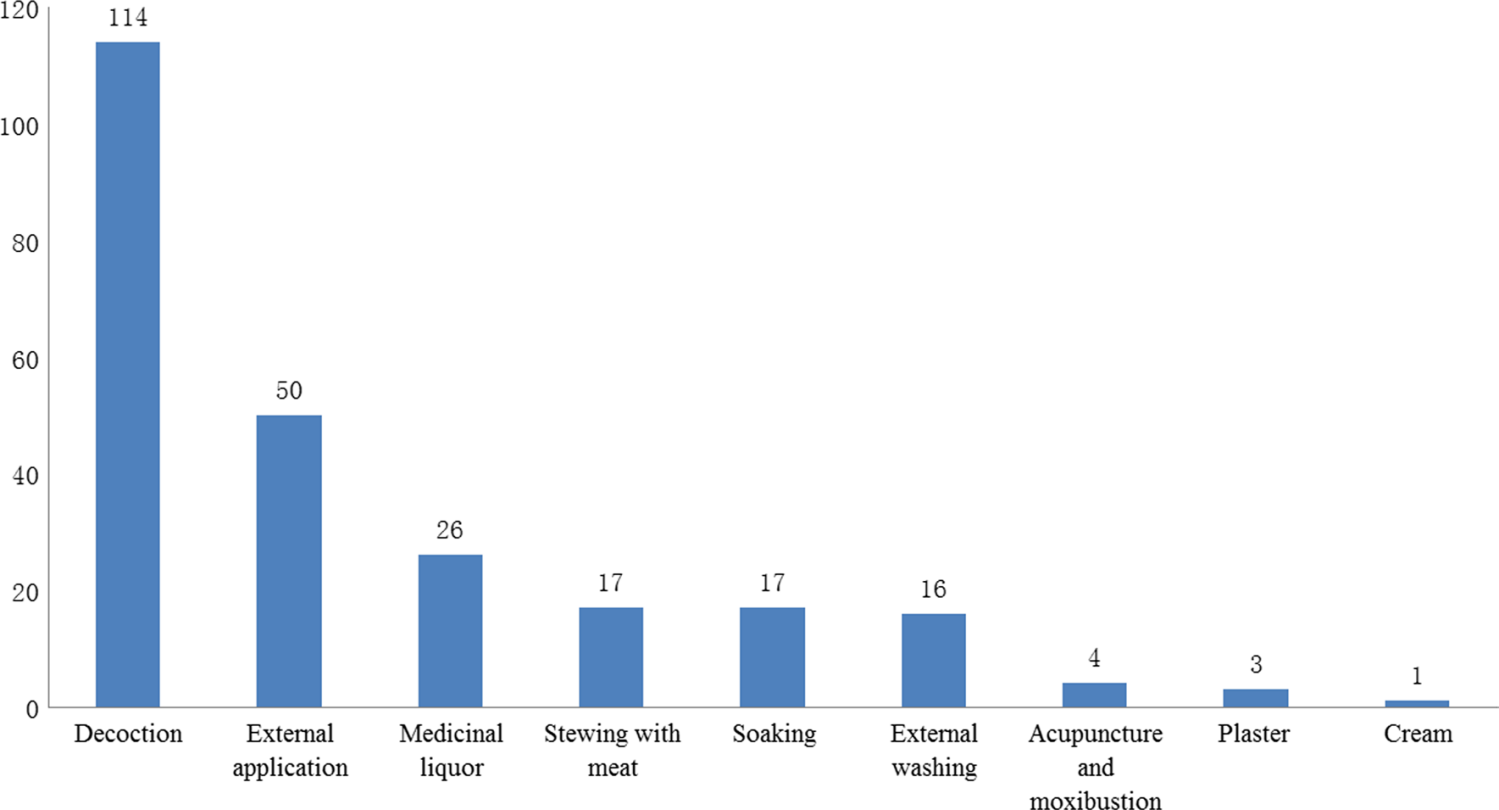
Treatment methods used by local healers in Gongcheng

In all, 248 prescriptions were collected through interviews with local healers; their methods of treatment mainly included nine types: decoction (114, 45.97%); external application (50, 20.16%); medicinal liquor (26, 10.48%); stewing with meat (17, 6.85%); soaking (17, 6.85%); external washing (16, 6.45%); acupuncture and moxibustion cupping (4, 1.61%); plaster (3, 1.21%); and cream (1, 0.40%) (Fig. 5). There are several methods of external application, including fresh herbs directly smashed for external application; dry herbs ground into a powder to spread on the affected area; or herbs mixed with tea oil, tung oil, vinegar, or secondary rice water for external application. Medicinal liquor has always been a preferred method by local healers, and many also prefer to use their secret recipes; medicinal liquor is easy to prepare, and its ingredients can be more effective in this form. It also has antiseptic and antitoxic effects, which can delay hydrolysis and enhance the stability of many drugs. During our investigation, we also found that some Yao medicine methods were more distinctive, for example, stewing herbs with pig tripe, pig feet, pig bones, snails, frogs, or fish.

Local healers believe that when fresh herbs are directly used for general decoction or external washing without being specially processed, their medicinal power is fierce and toxic side effects may occur, especially in the use of *Dayao* (a type of traditional medicine defined by local Yao people). When herbs are mixed with meat, bone, or other compatible stews, their power is lessened, and the toxicity of some fresh herbs can be reduced after prolonged decoction. The decoction is the most common method of herbal remedy preparation and is used widely by other ethnic groups [7, 8, 38–43]. In addition, a small number of local healers make plasters and creams, and some use moxibustion and cupping skills for treatment, but this is quite rare.

In the current study, the local healers used rosin, tung oil, or Huangdan and other auxiliary materials to make ointments such as rheumatic bone pain ointment, *Wuliu* ointment, and other commonly used ointments for the treatment of rheumatic bone pain, scalds, knife wounds, and other diseases. Furthermore, it is worth noting that local healers made *Liaodiaozhu* cream (a type of cream prepared mainly from the plant *Cynanchum paniculatum)*, wherein the crystals precipitate from the freshly picked herbs after washing, kneading, and sealing in a bottle. This cream is widely popular for its effect, convenience, and ease of preservation in treating common diseases such as knife wounds and styes.

### Diversity of medicinal plants used in the study area

In this investigation, 306 medicinal plant species were identified in 248 prescriptions of local healers in Gongcheng, belonging to 116 families and 255 genera. The results provided each species information, including scientific name, family, Chinese name, local name, habit, medicinal part, usage, and voucher specimen number (Table 4). The statistical analysis of families and species is shown in Table 5. At the family level, Asteraceae contained the most species (25 species), followed by the Fabaceae (17), Rubiaceae (12), Rutaceae (9), Rosaceae (8), Lamiaceae (8), Malvaceae (8), Polygonaceae (8), Vitaceae (7), and Primulaceae (6). Similar results have been shown in other areas of China, where many species belonged to these families [7, 27, 35, 36, 44–46]. These ten families accounted for 8.62% of the total number of families, but the number of species included accounted for 35.29% of the total number of species. Although there were many medicinal plants commonly used by local healers in different families, only a few families were highlighted. There were 49 families with 2–5 species, accounting for 42.24% of all families; the remaining 57 families contained only one species.

**Table 4:** Inventory of medicinal plants traditionally used by Yao people in Gongcheng

No.	Scientific name	Family	Chinese name	Local name	Life forms	Medicinal part	Usage	Voucher specimen number	FC	RFC
1	*Lycopodiastrum casuarinoides* (Spring) Holub ex R. D. Dixit	Lycopodiaceae	Teng shi song	Jin gu feng, Shen jin cao, Song jin teng	Herbaceous vine	Whole plant	Lumbocrural pain, falling injury	YY1567	1	0.004
2	*Selaginella doederleinii* Hieron	Selaginellaceae	Shen lv juan bai	Shi shang bai	Herb	Whole plant	Jaundice hepatitis	450332141115081LY	1	0.004
3	*Selaginella uncinata* (Desv. ex Poir.) Spring	Selaginellaceae	Cui yun cao	Cui yun cao	Herb	Whole plant	Gallstone, hepatitis	450332141117031LY	1	0.004
4	*Equisetum ramosissimum* Desf	Equisetaceae	Jie jie cao	Bi tong cao	Herb	Whole plant	Stone, constipation, epistaxis, scald, allergies	450332141117014LY	7	0.028
5	*Angiopteris fokiensis* Hieron	Marattiaceae	Fu jian guan yin zuo lian	Ma ti jue	Herb	Rhizome	Cooling blood, stop bleeding, relieve itching, analgesia	450332141115050LY	1	0.004
6	*Cibotium barometz* (L.) J.Sm	Cibotiaceae	Jin mao gou ji	Gou ji	Herb	Rhizome	Rheumatic bone pain, lumbar hyperplasia, paraplegia	450332141115049LY	1	0.004
7	*Alsophila spinulosa* (Wall. ex Hook.) R. M. Tryon	Cyatheaceae	Suoluo	Long gu feng	Herb	Stem	Insomnia, rheumatic bone pain, high fever, gynecological disorders	6–5029	2	0.008
8	*Lygodium japonicum* (Thunb.) Sw	Lygodiaceae	Hai jin sha	Jin sha teng	Herbaceous vine	Whole plant	Infertility, Stone	450332141114023LY	2	0.008
9	*Pteris multifida* Poir	Pteridaceae	Jing lan feng wei jue	Feng wei cao	Herb	Whole plant	Scald, furuncle, allergies	450332141114067LY	5	0.020
10	*Pteris semipinnata* L	Pteridaceae	Ban bian qi	Ban bian ju	Herb	Whole plant	Snake bite	YY1568	1	0.004
11	*Asplenium antrophyoides* Christ	Aspleniaceae	Xia chi chao jue	Zhen wu jian	Herb	Whole plant	Acute tonsillitis, bruise, undefined swelling and soreness	450332150401009LY	1	0.004
12	*Nephrolepis cordifolia* (L.) C. Presl	Nephrolepidaceae	Shen jue	Tian e bao dan	Herb	Tuber	Hyperthyroidism	450332150330026LY	1	0.004
13	*Humata griffithiana* (Hook.) C. Chr	Davalliaceae	Bei gai yin shi jue	Bai mao lian	Herb	Whole plant	Bruise, gynecological disorders, infantile malnutrition	450332150330012LY	1	0.004
14	*Lemmaphyllum drymoglossoides* (Baker) Ching	Polypodiaceae	Bao shi lian	Bao shi jue	Herb	Whole plant	Lung abscess, infantile malnutrition, liver cirrhosis	6090323	1	0.004
15	*Neolepisorus fortunei* (T. Moore) Li Wang	Polypodiaceae	Jiang nan xing jue	Sheng fa cao	Herb	Whole plant	Bald spot	450332141116062LY	1	0.004
16	*Pyrrosia lingua* (Thunb.) Farw	Polypodiaceae	Shi wei	Fei jing cao	Herb	Whole plant	Lung abscess	450332141116055LY	1	0.004
17	*Drynaria roosii Nakaike*	Polypodiaceae	Hujue	Gu sui bu	Herb	Rhizome	Bruise, fracture	450332141117067LY	7	0.028
18	*Cycas revoluta* Thunb	Cycadaceae	Su tie	Tie shu hua	Shrub	Flower, Seed	Male flowers are used for tonifying Yang, enteritis, female flowers are used for gynecological disorders, hepatitis, stomachache	YY1569	3	0.012
19	*Ginkgo biloba* L	Ginkgoaceae	Yin xing	Bai guo	Tree	Seed	cough, frequent urination, abnormal leukorrhea	450332150414035LY	1	0.004
20	*Pinus massoniana* Lamb	Pinaceae	Ma wei song	Song zhen	Tree	Leaf, Bark	Rheumatic bone pain	450332150330019LY	3	0.012
21	*Cunninghamia lanceolata* (Lamb.) Hook	Cupressaceae	Shan mu	Shan shu	Tree	Stem and leaf	Stone, rubella	YY1570	1	0.004
22	*Gnetum parvifolium* (Warb.) Chun	Gnetaceae	Xiao ye mai ma teng	Ma gu feng	Woody vine	Stem	Rheumatic bone pain, stroke	450332150615025LY	4	0.016
23	*Houpoea officinalis* (Rehder & E. H. Wilson) N. H. Xia & C. Y. Wu	Magnoliaceae	Hou pu	Hou po	Tree	Bark, Flower	Stem bark are used for thick tongue fur, cirrhosis ascites, infantile diarrhea, infantile malnutrition, Flower are used for angina	6090388	2	0.008
24	*Kadsura coccinea* (Lem.) A. C. Sm	Schisandraceae	Hei lao hu	Da zuan	Woody vine	Stem	Rheumatic bone pain, traumatic injury, knuckles swollen and painful	450332150617044LY	4	0.016
25	*Kadsura heteroclita* (Roxb.) Craib	Schisandraceae	Yi xing nan wu wei zi	Hai feng teng	Woody vine	Stem	Rheumatic bone pain	142	1	0.004
26	*Kadsura longipedunculata* Finet & Gagnep	Schisandraceae	Nan wu wei zi	Xiao zuan	Woody vine	Stem	Rheumatism, stomachache, gastric hemorrhage	450332150331002LY	14	0.056
27	*Kadsura oblongifolia* Merr	Schisandraceae	Leng fan teng	Xiao hong zuan	Woody vine	Stem	Rheumatism, hyperostosis, sciatica, stroke sequela	YY1571	1	0.004
28	*Fissistigma oldhamii* (Hemsl.) Merr	Annonaceae	Gua fu mu	Tie zuan, Xun gu feng	Shrub	Stem, Root	Tocolysis, rheumatic bone pain, sciatica, fever, typhoid	450332141116001LY	2	0.008
29	*Cinnamomum camphora* (L.) J. Presl	Lauraceae	Zhang	Zhang shu, Xiang zhang	Tree	Stem and leaf	Bruise, bee sting	450332141114020LY	3	0.012
30	*Cinnamomum cassia* J. Presl	Lauraceae	Rou gui	Gui zhi, Gui pi	Tree	Bark, Stem and leaf	Renal elimination of water, rheumatic bone pain, stroke, cold	YY1572	4	0.016
31	*Litsea cubeba* (Lour.) Pers	Lauraceae	Shan ji jiao	Shan cang zi	Tree	Fruit, Root	Scapulohumeral periarthritis, abdominal distension pain, rheumatic bone pain, undefined swelling and pain	450332141115083LY	3	0.012
32	*Aconitum carmichaeli* Debx	Ranunculaceae	Wu tou	Cao wu	Herb	Root	Dispel wind-damp, bruise, hyperostosis	YY1573	10	0.040
33	*Clematis chinensis* Osbeck	Ranunculaceae	Wei ling xian	Hei jiu niu	Woody vine	Root	Bruise, rheumatic bone pain, hyperostosis, fallopian tube obstruction	450332141114071LY	7	0.028
34	*Ranunculus sieboldii* Miq	Ranunculaceae	Yang zi mao gen	Ya jiao cai	Herb	Whole plant	Bruise, eye inflammation	450332150329004LY	1	0.004
35	*Nuphar pumila* (Timm) DC	Nymphaeaceae	Ping peng cao	Leng gu feng	Herb	Rhizome	Pneumonia cough, rheumatic bone pain, uterine cold, dysmenorrhea, amenorrhea	6–5218	3	0.012
36	*Dysosma versipellis* (Hance) M. Cheng	Berberidaceae	Ba jiao lian	Du jiao lian	Herb	Rhizome	Hemorrhoids, undefined swelling and soreness, lymphadenopathy, snake bite	6090016	1	0.004
37	*Mahonia fortunei* (Lindl.) Fedde	Berberidaceae	Shi da gong lao	Tu huang lian	Shrub	Flower	Hepatitis	450332141118024LY	2	0.008
38	*Akebia trifoliata* (Thunb.) Koidz	Lardizabalaceae	San ye mu tong	Mu tong, Lan jiu niu	Woody vine	Stem	Stone, rheumatism paralysis, fallopian tube obstruction, enteroptosis, hepatitis,liver ascites, hemorrhoids	450332150330025LY	8	0.032
39	*Sargentodoxa cuneata* (Oliv.) Rehder & E. H. Wilson	Lardizabalaceae	Da xue teng	BinLang zuan	Woody vine	Stem	Hyperostosis, rheumatic bone pain, traumatic injury	450332150821046LY	4	0.016
40	*Cyclea hypoglauca* (Schauer) Diels	Menispermaceae	Fen ye lun huan teng	Shan dou gen, Jin xian feng	Herbaceous vine	Root	Lymphadenitis, cough, toothache, acute pharyngitis, typhoid dysentery	450332150614022LY	5	0.020
41	*Tinospora sagittata* (Oliv.) Gagnep	Menispermaceae	Qing niu dan	Jin guo lan	Herbaceous vine	Root tuber	Detumescence, cooling blood, sore and ulcer, undefined swelling and soreness, mastitis, traumatic injury, stomachache, toothache, lymphadenitis, gynecological inflammation, gynecological cyst	6090322	1	0.004
42	*Tinospora sinensis* (Lour.) Merr	Menispermaceae	Zhong hua qing niu dan	Kuan jin teng	Woody vine	Stem	Dispel wind, analgesia, relaxing sinew and activating coll	450332151022002LY	1	0.004
43	*Aristolochia debilis* Sieb. et Zucc	Aristolochiaceae	Ma dou ling	Qing mu xiang	Herbaceous vine	Root tuber	herpes zoster	YY1574	1	0.004
44	*Aristolochia tubiflora* Dunn	Aristolochiaceae	Guan hua ma dou ling	Tong cheng hu, Tian ran cao	Herbaceous vine	Root tuber	Analgesia, stop bleeding, relieving superficies, febrile convulsion, snake bite	450332160516008LY	1	0.004
45	*Aristolochia gongchengensis* Y.S.Huang,Y.D.Peng & C.R.Lin	Aristolochiaceae	Gong cheng ma dou ling	Tian zuan	Woody vine	Root tuber	Stroke, thrombus, dizziness, headache	Y3030	1	0.004
46	*Asarum insigne* Diels	Aristolochiaceae	Jin er huan	Tu xi xin	Herb	Root	Snake bite, stomachache	YY1575	1	0.004
47	*Houttuynia cordata* Thunb	Saururaceae	Jicai	Yu xing cao	Herb	Whole plant	Wind-heat type common cold	450332160512010LY	2	0.008
48	*Saururus chinensis* (Lour.) Baill	Saururaceae	San bai cao	Guo tang ou	Herb	Rhizome	Dysmenorrhea, gynecological inflammation, dissipate blood stasis, stimulate saliva, cold cough	450332150616043LY	4	0.016
49	*Piper wallichii* (Miq.) Hand.-Mazz	Piperaceae	Shi nan teng	Shi nan feng	Herbaceous vine	Whole plant	Gout	450332160512006LY	1	0.004
50	*Sarcandra glabra* (Thunb.) Nakai	Chloranthaceae	Cao shan hu	Zhong jie feng, Jiu jie cha	Shrub	Whole plant	Rheumatic bone pain	450332141115031LY	3	0.012
51	*Macleaya cordata* (Willd.) R. Br	Papaveraceae	Bo luo hui	Hao tong geng	Herb	Whole plant	Traumatic injury	450332141116045LY	1	0.004
52	*Viola inconspicua* Blume	Violaceae	Chang ejincai	Li tou cao	Herb	Whole plant	Typhoid	450332150412015LY	1	0.004
53	*Viola philippica* Cav	Violaceae	Zi hua di ding	Li tou cao	Herb	Whole plant	Pinkeye, undefined swelling and soreness	450332150412018LY	1	0.004
54	*Polygala fallax* Hemsl	Polygalaceae	Huang hua dao shui lian	Huang hua shen	Shrub	Root	enrich the blood, tonifying qi, rheumatic bone pain, male infertility	450332150617027LY	8	0.032
55	*Polygala polifolia* C. Presl	Polygalaceae	Xiao hua yuan zhi	Gua zi lian	Herb	Whole plant	Infantile malnutrition, traumatic injury, hyperostosis	450332150910001LY	1	0.004
56	*Saxifraga stolonifera* Curtis	Saxifragaceae	Hu er cao	Jin xian diao furong	Herb	Whole plant	Otitis media, sore and ulcer	YY1576	1	0.004
57	*Drosera peltata* Thunb	Droseraceae	Mao gao cai	Di xia ming zhu, Yi li jin dan	Herb	Corm	Insecticide, relieve itching, hyperostosis	YY1577	1	0.004
58	*Portulaca oleracea* L	Portulacaceae	Ma chi xian	Gua zi cai	Herb	Whole plant	Dysentery, herpes zoster	450332150908001LY	2	0.008
59	*Talinum paniculatum* (Jacq.) Gaertn	Talinaceae	Tu ren shen	Tu ren shen	Herb	Root	Hemorrhoids	450332141115024LY	1	0.004
60	*Antenoron filiforme* (Thunb.) Roberty & Vautier	Polygonaceae	Jin xian cao	Man jing feng	Herb	Whole plant	Stone	450332150619003LY	1	0.004
61	*Fagopyrum dibotrys* (D. Don) H. Hara	Polygonaceae	Jin qiao mai	Ye qiaomai	Herb	Whole plant	Long menstrual period, prostatitis	450332141115116LY	2	0.008
62	*Fallopia multiflora* (Thunb.) Haraldson	Polygonaceae	He shou wu	Shou wu	Herbaceous vine	Stem, Root tuber	Furuncle, bruise, gray hair	450332141114078LY	2	0.008
63	*Polygonum chinense* L	Polygonaceae	Huo tan mu	Chi di li	Herb	Whole plant	Burn and scald, furuncle, insecticide, relieve itching, eye inflammation	450332141115051LY	5	0.020
64	*Polygonum hydropiper* L	Polygonaceae	Shui liao	La liao	Herb	Whole plant	Diarrhea, acute gastroenteritis	YY1578	1	0.004
65	*Polygonum perfoliatum* L	Polygonaceae	Gang ban gui	She bu guo	Herbaceous vine	Whole plant	Furuncle, infantile malnutrition	450332150411010LY	2	0.008
66	*Polygonum runcinatum* var. *sinense* Hemsl	Polygonaceae	Chi jingsan	Qian jin zi	Herb	Rhizome	Snake bite, sciatica, stomachache, traumatic injury, undefined swelling and soreness, mastitis	450332151020001LY	2	0.008
67	*Reynoutria japonica* Houtt	Polygonaceae	Hu zhang	Yin yang lian	Herb	Rhizome	Constipation, liver cirrhosis, hepatitis, liver ascites, cough	450332150331016LY	5	0.020
68	*Phytolacca acinosa* Roxb	Phytolaccaceae	Shang lu	Shan luo bo	Herb	Root	Inflammation, high fever, dispel wind and damp, bruise, hemorrhoids	450332150401021LY	1	0.004
69	*Achyranthes aspera* L	Amaranthaceae	Tu niu xi	Niu xi feng, Dao kou cao	Herb	Root	Hyperostosis, rheumatic bone pain, dysmenorrhea, hemorrhoids	450332150821023LY	12	0.048
70	*Amaranthus spinosus* L	Amaranthaceae	Ci xian	Ci xiancai	Herb	Whole plant	Hemorrhoids	450332141114056LY	2	0.008
71	*Celosia argentea* L	Amaranthaceae	Qing xiang	Qing xiang zi	Herb	Seed	Eye inflammation	450332141114004LY	1	0.004
72	*Celosia cristata* L	Amaranthaceae	Ji guan hua	Ji guan hua	Herb	Flower	gynecological inflammation	YY1579	1	0.004
73	*Anredera cordifolia* (Ten.) Steenis	Basellaceae	Luo kui shu	Teng san qi	Herbaceous vine	Bulbil	Infantile malnutrition	450332150820046LY	2	0.008
74	*Edgeworthia chrysantha* Lindl	Thymelaeaceae	Jie xiang	Bao nuan feng	Shrub	Whole plant	Postpartum persistent lochia, infertility, dispel cold	YY1580	2	0.008
75	*Wikstroemia indica* (L.) C. A. Mey	Thymelaeaceae	Liao ge wang	Di shi liu	Shrub	Leaf, Root	Furuncle, snake bite	450332141116064LY	2	0.008
76	*Pittosporum pauciflorum* Hook. & Arn	Pittosporaceae	Shao hua hai tong	Shang shan hu	Shrub	Root	Bruise, rheumatic bone pain, lumbar disc herniation	450332141116054LY	1	0.004
77	*Gynostemma pentaphyllum* (Thunb.) Makino	Cucurbitaceae	Jiao gu lan	Pan wang cha	Herbaceous vine	Whole plant	Stone	450332141117058LY	1	0.004
78	*Solena heterophylla* Lour	Cucurbitaceae	Mao gua	Lao shu ban gua	Herbaceous vine	Root,Whole plant	Hyperthyroidism, undefined swelling and soreness, gynecological cyst	450332150614032LY	3	0.012
79	*Begonia fimbristipula* Hance	Begoniaceae	Zi bei tian kui	San xue zi	Herb	Whole plant	Pyogenic infections, bruise, rheumatic bone pain	450332150330021LY	1	0.004
80	*Begonia longifolia* Blume	Begoniaceae	Cu hui qiu hai tang	Rou ban bian lian	Herb	Whole plant	Sphagitis	450332150412032LY	1	0.004
81	*Camellia sinensis* (L.) O. Kuntze	Theaceae	Cha	Cha ye	Shrub	Leaf	Vomiting and diarrhea	450332141115041LY	3	0.012
82	*Melastoma dodecandrum* Lour	Melastomataceae	Di nie	Di pu tao	Shrub	Whole plant	Infantile malnutrition, diarrhea, bone injury	450332150820057LY	1	0.004
83	*Osbeckia crinita* Benth. ex C.B. Clarke	Melastomataceae	Jia chao tian guan	Tian pu tao	Shrub	Whole plant	Tooth decay, bone injury, cold, infertility, rectocele	450332150820010LY	1	0.004
84	*Hypericum japonicum* Thunb. ex Murray	Hypericaceae	Di er cao	Tian ji huang	Herb	Whole plant	Liver cirrhosis, typhoid, hepatitis	450332150614003LY	4	0.016
85	*Hypericum sampsonii* Hance	Hypericaceae	Yuan bao cao	Fan chuan cao	Herb	Whole plant	Fallopian tube obstruction, dysmenorrhea, puerperal cold	YY1581	1	0.004
86	*Helicteres angustifolia* L	Malvaceae	Shan zhi ma	Ye you ma	Shrub	Root	Influenza, typhoid, clearing summer-heat	YY1582	2	0.008
87	*Abelmoschus moschatus* Medik	Malvaceae	Huang kui	Ye mian hua, Huang shu kui	Herb	Seed, Root	Stone, scald, snake bite, scabies	450332150823014LY	3	0.012
88	*Hibiscus mutabilis* L	Malvaceae	Mu furong	Fu rong hua	Shrub	Leaf	Snake bite	450332141114066LY	1	0.004
89	*Hibiscus syriacus* L	Malvaceae	Mu jin	Cha lihua	Shrub	Root	Amenorrhea, leukorrhagia, maleinfertility	6090364	3	0.012
90	*Sida rhombifolia* L	Malvaceae	Bai bei huang hua ren	Huang hua cao	Shrub	Whole plant	Furuncle	450332141114057LY	1	0.004
91	*Pterospermum heterophyllum* Hance	Malvaceae	Fan bai ye shu	Ban bian feng	Tree	Whole plant	Hemiplegia, set a broken bone, rheumatic bone pain	450332150331052LY	1	0.004
92	*Urena lobata* L	Malvaceae	Di tao hua	Di tao hua	Shrub	Whole plant	Dysmenorrhea, long menstrual period, amenorrhea, infertility, typhoid	450332141117023LY	6	0.024
93	*Urena procumbens* L	Malvaceae	Fantian hua	Gou jiao ji	Shrub	Whole plant	Typhoid	402776	1	0.004
94	*Acalypha australis* L	Euphorbiaceae	Tie xiancai	Bang ke cai	Herb	Whole plant	Epistaxis, infantile malnutrition, dysentery	450332141114040LY	2	0.008
95	*Croton tiglium* L	Euphorbiaceae	Ba dou	Ba bai li	Shrub	Leaf	Lower body cold, snake bite, arthritis, purgation, disperse accumulations	YY1583	3	0.012
96	*Euphorbia humifusa* Willd. ex Schltdl	Euphorbiaceae	Di jin	Nai jiang cao	Herb	Whole plant	Infantile malnutrition, gastroenteritis, diarrhea	450332150615016LY	1	0.004
97	*Mallotus apelta* (Lour.) Müll. Arg	Euphorbiaceae	Bai bei ye	Bai bei tong	Shrub	Root-bark	Rectocele, leukorrhagia	450332141115091LY	3	0.012
98	*Ricinus communis* L	Euphorbiaceae	Bi ma	Bi ma	Shrub	Seed	Iron injury, suppuration	450332141115020LY	2	0.008
99	*Glochidion puberum* (L.) Hutch	Phyllanthaceae	Suan pan zi	Hong mao man tou guo	Shrub	Whole plant	Fallopian tube obstruction	450332150614024LY	1	0.004
100	*Phyllanthus urinaria* L	Phyllanthaceae	Ye xia zhu	Ye xia zhu	Herb	Whole plant	Hepatitis, improving eyesight, antidiarrheal	450332151021001LY	2	0.008
101	*Dichroa febrifuga* Lour	Hydrangeaceae	Chang shan	Ru gu feng	Shrub	Root	Dysmenorrhea, hepatitis, rheumatic bone pain	450332150619019LY	1	0.004
102	*Agrimonia pilosa* Ledeb	Rosaceae	Long ya cao	Xian he cao	Herb	Whole plant	Epistaxis, stomachache, enteritis, gynecological disorders hemorrhage, cold	450332150619012LY	2	0.008
103	*Amygdalus persica* L	Rosaceae	Tao	Tao reng	Tree	Seed	Stone, hyperostosis, stroke, bruise	6090361	4	0.016
104	*Duchesnea indica* (Andr.) Focke	Rosaceae	She mei	She pao le	Herb	Root	Dysentery, herpes zoster, undefined swelling and soreness	450332150401037LY	1	0.004
105	*Eriobotrya japonica* (Thunb.) Lindl	Rosaceae	Pi pa	Pi pa ye	Tree	Leaf	Wind-heat type common cold	450332150329034LY	1	0.004
106	*Malus doumeri* (Bois) A. Chev	Rosaceae	Tai wan lin qin	Da guo shan zha	Tree	Fruit	Diabetes	450332150907001LY	1	0.004
107	*Potentilla fragarioides* L	Rosaceae	Meiye wei ling cai	Di yang mei	Herb	Whole plant	Long menstrual period	YY1584	1	0.004
108	*Rosa laevigata* Michx	Rosaceae	Jin ying zi	Tang ci guo	Shrub	Root, Stem	Furuncle, scald	450332150331062LY	1	0.004
109	*Sanguisorba officinalis* L	Rosaceae	Di yu	Ma liu an	Herb	Root	Bruise, infantile diarrhea, scald, hemorrhoids, enteritis, stomachache, stone	YY1585	12	0.048
110	*Abrus cantoniensis* Hance	Fabaceae	Guang zhou xiang si zi	Ji gu cao	Shrub	Whole plant	Hepatitis	YY1586	2	0.008
111	*Bauhinia championii* (Benth.) Benth	Fabaceae	Long xu teng	Jiu long zuan	Woody vine	Stem	Rheumatic bone pain, gout, set a broken bone	450332141114062LY	3	0.012
112	*Canavalia gladiata* (Jacq.) DC	Fabaceae	Dao dou	Dao dou jia	Herb	Legume	Costalgia	YY1587	1	0.004
113	*Crotalaria albida* B. Heyne ex Roth	Fabaceae	Xiang ling dou	Huang hua di ding	Herb	Whole plant	Breast cancer, liver cancer	450332141117002LY	1	0.004
114	*Flemingia prostrata* Roxb. f. ex Roxb	Fabaceae	Qian jin ba	Tao ma zhuang	Shrub	Root	Hyperostosis, rheumatic bone pain, set a broken bone, eczema	6090270	12	0.048
115	*Gleditsia sinensis* Lam	Fabaceae	Zao jia	Zao ci	Tree	Legume, Thorn	Hemorrhoids, fallopian tube obstruction, rheumatic bone pain	371	4	0.016
116	*Kummerowia striata* (Thunb.) Schindl	Fabaceae	Ji yan cao	Ren zi cao	Herb	Whole plant	Vomiting and diarrhea, dog bite, milk accumulation in infants	450332150819024LY	5	0.020
117	*Lespedeza cuneata* (Dum.-Cours.) G. Don	Fabaceae	Jie ye tie sao zhou	Chuan yu liu	Shrub	Whole plant	Lumbago, diarrhea, stone	450332141114007LY	1	0.004
118	*Mucuna lamellata* Wilmot-Dear	Fabaceae	Zhepi li dou	Guo shan feng	Woody vine	Root	Hyperostosis	YY1588	1	0.004
119	*Ohwia caudata* (Thunb.) H. Ohashi	Fabaceae	Xiao huai hua	E ma huang	Shrub	Whole plant	Infantile diarrhea, infantile malnutrition, infantile dyspepsia, eye inflammation	450332141117012LY	5	0.020
120	*Phyllodium pulchellum* (L.) Desv	Fabaceae	Pai qian shu	Pai qian cao, Qian chuan mu	Shrub	Stem and leaf	Stomachache	450332141116080LY	1	0.004
121	*Pueraria montana* var. *lobata* (Willd.) Maesen & S. M. Almeida ex Sanjappa & Predeep	Fabaceae	Ge	Ge geng, Wu ceng feng	Herbaceous vine	Root	Cold, hyperostosis	450332151022001LY	4	0.016
122	*Senna occidentalis* (L.) Link	Fabaceae	Wang jiang nan	Ye guan men	Shrub	Root	Hyperthyroidism	450332141114077LY	2	0.008
123	*Senna tora* (L.) Roxb	Fabaceae	Jue ming	Cao jue ming	Herb	Seed, Leaf	Infantile malnutrition, stone, eye inflammation, rheumatic bone pain, scald	450332141114079LY	7	0.028
124	*Sophora flavescens* Aiton	Fabaceae	Ku shen	Ku shen	Herb	Root	Cervical erosion	450332160516005LY	1	0.004
125	*Spatholobus suberectus* Dunn	Fabaceae	Mi hua dou	Ji xue teng, Jiu ceng feng	Woody vine	Stem	Stroke, rheumatic bone pain	1	3	0.012
126	*Tadehagi triquetrum* (L.) H. Ohashi	Fabaceae	Hu lu cha	Hu lu zuan	Shrub	Whole plant	Liver cirrhosis, hepatitis	6090251	1	0.004
127	*Liquidambar formosana* Hance	Altingiaceae	Feng xiang shu	Lu lu tong	Tree	Fruit	Fallopian tube obstruction	450332150411044LY	1	0.004
128	*Semiliquidambar cathayensis* Hung T.Chang	Altingiaceae	Ban feng he	Ban he feng	Tree	Bark, Stem and leaf	Hyperostosis, rheumatic bone pain, lumbocrural pain, stroke	450332160515017LY	6	0.024
129	*Eucommia ulmoides* Oliv	Eucommiaceae	Du zhong	Du zhong	Tree	Bark, Stem and leaf	Rheumatic bone pain, hyperostosis, hypertension, alopecia	450332150907002LY	6	0.024
130	*Castanea mollissima* Blume	Fagaceae	Li	Ban li ye	Tree	Leaf	Tuberculosis	YY1589	1	0.004
131	*Ficus carica* L	Moraceae	Wu hua guo	Wu hua guo	Shrub	Root	Hemorrhoids	YY1590	1	0.004
132	*Ficus hirta* Vahl	Moraceae	Cu ye rong	Wu zhi niu nai	Shrub	Root	Infantile malnutrition, nephritis, abnormal leukorrhea, lack of milk after childbirth	450332141114019LY	2	0.008
133	*Ficus sarmentosa* var. *lacrymans* (H. Lév.) Corner	Moraceae	Wei jian pa teng rong	Jian ye rong	Shrub	Stem and leaf	Knife wound	450332141117036LY	1	0.004
134	*Morus alba* L	Moraceae	Sang	Sang bai pi	Tree	Root, Root-bark, Stem and leaf	Furuncle, hands and feet pain, gray hair	450332150410018LY	4	0.016
135	*Boehmeria nivea* (L.) Gaudich	Urticaceae	Zhu ma		Shrub	Root	Long menstrual period, furuncle	450332141114036LY	1	0.004
136	*Humulus scandens* (Lour.) Merr	Cannabaceae	lv cao	Wu zhua long	Herb	Whole plant	Herpes zoster, undefined swelling and soreness, sphagitis	450332141118008LY	1	0.004
137	*Ilex asprella* (Hook. & Arn.) Champ. ex Benth	Aquifoliaceae	Cheng xing shu	Bai jie mu	Shrub	Root, Leaf	High fever, analgesia, typhoid, hepatitis, liver ascites, tuberculosis, traumatic injury	YY1591	1	0.004
138	*Ilex chinensis* Sims	Aquifoliaceae	Dong qing	Si ji qing	Tree	Stem	Furuncle, knife wound	403083	1	0.004
139	*Ilex pubescens* Hook. & Arn	Aquifoliaceae	Mao dong qing	Da bai jie, Bai jie dou	Shrub	Root	Sphagitis, hypertension, furuncle, traumatic injury	450332141115122LY	1	0.004
140	*Ilex rotunda* Thunb	Aquifoliaceae	Tie dong qing	Jiu bi ying	Tree	Bark	Breast cancer, liver cancer, infantile high fever, sphagitis	450332141115075LY	2	0.008
141	*Euonymus fortunei* (Turcz.) Hand.-Mazz	Celastraceae	Fu fang teng	Guo qiang feng	Woody vine	Whole plant	Rectocele, enteritis, anemia, infantile malnutrition	450332150330013LY	3	0.012
142	*Mappianthus iodoides* Hand.-Mazz	Icacinaceae	Ding xin teng	Tong zuan	Woody vine	Stem	Rheumatic bone pain, stroke, acute filthy disease, cold	YY1592	5	0.020
143	*Taxillus chinensis* (DC.) Danser	Loranthaceae	Guang ji sheng	Sang ji sheng	Shrub	Stem and leaf	Rheumatism, stone	YY1593	1	0.004
144	*Rhamnus crenata* Sieb. et Zucc	Rhamnaceae	Chang ye dong lv	Ku li gen	Shrub	Root	Skin diseases, furuncle	0101	1	0.004
145	*Ventilago leiocarpa* Benth	Rhamnaceae	Yi he guo	Zi jiu niu	Woody vine	Root	Syphilis, amenorrhea, long menstrual period, fallopian tube obstruction, breast cancer, liver cancer, rheumatic bone pain	450332160603001LY	7	0.028
146	*Elaeagnus glabra* Thunb	Elaeagnaceae	Man hu tui zi	Yang nai guo	Shrub	Root, Leaf	Epilepsy	450332141117041LY	1	0.004
147	*Ampelopsis grossedentata* (Hand.-Mazz.) W. T. Wang	Vitaceae	Xian chi she pu tao	Teng cha, Tian cha	Woody vine	Stem and leaf	Hyperglycemia, hyperlipemia	450332150619053LY	2	0.008
148	*Ampelopsis japonica* (Thunb.) Makino	Vitaceae	Bai lian	Jiu zi niang niang	Woody vine	Root tuber	Hyperthyroidism	450332160511015LY	2	0.008
149	*Cayratia pseudotrifolia* W.T.Wang	Vitaceae	Wu lianmei	Zhu po teng	Herbaceous vine	Stem and leaf	Cellulitis, undefined swelling and soreness	450332141118009LY	1	0.004
150	*Cissus assamica* (M. A. Lawson) Craib	Vitaceae	Ku lang teng	Hong bei si chou	Woody vine	Root	Rheumatism, bruise, snake bite, furuncle, osteomyelitis	6–5317	1	0.004
151	*Cissus pteroclada* Hayata	Vitaceae	Yi jing bai fen teng	Si fang zuan	Herbaceous vine	Stem	Warm limbs meridian, rheumatic bone pain	YY1594	2	0.008
152	*Tetrastigma hemsleyanum* Diels & Gilg	Vitaceae	San ye ya pa teng	San ye qing, Shi hou zi	Herbaceous vine	Root tuber	Dysmenorrhea, amenorrhea, lymphadenitis, hyperthyroidism, stone, lymphadenopathy	450332150415018LY	10	0.040
153	*Tetrastigma planicaule* (Hook. f.) Gagnep	Vitaceae	Bian dan teng	Bian gu feng	Woody vine	Stem	Rheumatism, relaxing sinew and activating coll	6–5242	1	0.004
154	*Citrus maxima* (Burm.) Merr	Rutaceae	You	Yong zi ke	Tree	Rind	Fallopian tube obstruction	YY1595	1	0.004
155	*Citrus reticulata* Blanco	Rutaceae	Gan ju	Chen pi	Tree	Rind, Stem	Hemorrhoids, cough	450332141116034LY	2	0.008
156	*Citrus trifoliata* L	Rutaceae	Zhi	Zhi ke, Zhi shi	Tree	Fruit	Fallopian tube obstruction, stroke, bad urination and defecation	450332150819037LY	3	0.012
157	*Melicope pteleifolia* (Champ. ex Benth.) T. G. Hartley	Rutaceae	San ya ku	San cha ku	Tree	Root, Leaf	Typhoid	450332150331035LY	1	0.004
158	*Phellodendron chinense* var. *glabriusculum* Schneid	Rutaceae	Tu ye huang bo	Huang bo	Tree	Bark	Snake bite, numbness and distension of feet, hemorrhoids	YY1596	4	0.016
159	*Tetradium ruticarpum* (A. Juss.) T. G. Hartley	Rutaceae	Wu zhu yu	Cha la	Tree	Leaf, Fruit	Toothache, cold, typhoid	450332141116041LY	3	0.012
160	*Toddalia asiatica* (L.) Lam	Rutaceae	Fei long zhang xue	Zou xue feng	Woody vine	Root	Analgesia, rheumatism, bruise, fracture	450332141116032LY	2	0.008
161	*Zanthoxylum armatum* DC	Rutaceae	Zhu ye hua jiao	Tu hua jiao	Shrub	Whole plant	Toothache, sciatica, hyperostosis	450332141117043LY	2	0.008
162	*Zanthoxylum austrosinense* C. C. Huang	Rutaceae	Ling nan hua jiao	Sou shan hu	Shrub	Whole plant	Hyperostosis, rheumatism, bruise	YY1597	3	0.012
163	*Picrasma quassioides* (D. Don) Benn	Simaroubaceae	Ku shu	Tai ban jiu	Tree	Stem	Tuberculosis, constipation	6–5229	3	0.012
164	*Sabia japonica* Maxim	Sabiaceae	Qing feng teng	Yi ci liang zui, Liang zui ci	Woody vine	Stem	Bruise, set a broken bone, rheumatic bone pain, hyperostosis	450332150401047LY	7	0.028
165	*Rhus chinensis* Mill	Anacardiaceae	Yan fu mu	Pao mu shu	Tree	Root	Detumescence, snake bite, traumatic injury	450332150820021LY	1	0.004
166	*Toxicodendron sylvestre* (Sieb. et Zucc.) Kuntze	Anacardiaceae	Mu la shu	Shan qi shu	Tree	Leaf	Knife wound, traumatic injury, epilepsy	450332150614044LY	1	0.004
167	*Alangium chinense* (Lour.) Harms	Cornaceae	Ba jiao feng	Bai long xu	Tree	Root	Typhoid	450332150617031LY	1	0.004
168	*Aralia spinifolia* Merr	Araliaceae	Chang ci song mu	Bai niao bu zhan	Shrub	Root	Cold, typhoid, eczema	450332150819036LY	2	0.008
169	*Eleutherococcus nodiflorus* (Dunn) S. Y. Hu	Araliaceae	Xi zhu wu jia	Wu jia pi	Shrub	Root-bark	Rheumatic bone pain, numbness and distension of feet, red and swollen eyes, hyperostosis, detumescence, analgesia	450332151016007LY	7	0.028
170	*Eleutherococcus trifoliatus* (L.) S. Y. Hu	Araliaceae	Bai le	San ye wu jia	Shrub	Root-bark	Stone, wind-heat type common cold, dampness-heat in lower jiao, dysentery	450332141114016LY	1	0.004
171	*Schefflera heptaphylla* (L.) Frodin	Araliaceae	E zhang chai	Ya jiao mu	Shrub	Root	Cold, typhoid, rheumatic bone pain, ankle pain, stroke, acute hepatitis, liver ascites, psychosis	450332141115085LY	14	0.056
172	*Tetrapanax papyrifer* (Hook.) K. Koch	Araliaceae	Tong tuo mu	Yao ying feng	Shrub	Stem pith	Costalgia	402880	1	0.004
173	*Angelica decursiva* (Miq.) Franch. & Sav	Apiaceae	Zi hua qian hu	Qian hu	Herb	Root	Hemorrhoids, hyperthyroidism	450332150821043LY	1	0.004
174	*Bupleurum marginatum* Wall. ex DC	Apiaceae	Zhu ye chai hu	Nan chai hu	Herb	Whole plant	Wind-heat type common cold, typhoid, hepatitis B, hemorrhoids	450332160511014LY	9	0.036
175	*Centella asiatica* (L.) Urb	Apiaceae	Ji xue cao	Lei gong gen	Herb	Whole plant	Antiemetic, antidiarrheal, traumatic injury, scald by hot water and fire, stone	YY1598	2	0.008
176	*Ostericum citriodorum* (Hance) C. Q. Yuan & R. H. Shan	Apiaceae	Ge shan xiang	Xiang bai zhi	Herb	Root	Hyperthyroidism, ileus, analgesia, relieve itching	450332150614052LY	1	0.004
177	*Gaultheria leucocarpa* var. *yunnanensis* (Franch.) T. Z. Hsu & R. C. Fang	Ericaceae	Dian bai zhu	Xia shan hu, Man shan xiang	Shrub	Whole plant	Bruise, rheumatic bone pain	450332160512020LY	1	0.004
178	*Rhododendron molle* (Blume) G. Don	Ericaceae	Yang zhi zhu	Mao lao hu, San qian san	Shrub	Root	Rheumatism, bruise, stone, hyperostosis	YY1599	2	0.008
179	*Symplocos paniculata* (Thunb.) Miq	Symplocaceae	Bai tan		Shrub	Root	Hepatitis, bone injury, leukorrheal diseases, knife wound	450332150614028LY	2	0.008
180	*Gelsemium elegans* (Gardner & Champ.) Benth	Gelsemiaceae	Gou wen	Duan chang cao	Woody vine	Root	Lumbago, rheumatism, set a broken bone	450332141115030LY	1	0.004
181	*Jasminum lanceolaria* Roxb	Oleaceae	Qing xiang teng	Po gu feng	Woody vine	Whole plant	Sequela of injury, rheumatic bone pain	YY1600	1	0.004
182	*Ligustrum lucidum* W. T. Aiton	Oleaceae	Nv zhen	Nv zhen zi	Tree	Fruit	Gray hair, alopecia	450332150908003LY	3	0.012
183	*Cynanchum corymbosum* Wight	Apocynaceae	Ci gua	Ge shan xiao, Shui yang liu	Herbaceous vine	Whole plant	Enteritis, stomachache, allergy, scald	450332141116076LY	4	0.016
184	*Cynanchum paniculatum* (Bunge) Kitag. ex H. Hara	Apocynaceae	Xu chang qing	Liao diao zhu	Herb	Whole plant	Knife wound, stye, psoriasis, bruise, poison bee sting, rheumatism, lymphadenopathy	YY1601	7	0.028
185	*Trachelospermum jasminoides* (Lindl.) Lem	Apocynaceae	Luo shi	Pa qiang feng	Woody vine	Stem	Infantile malnutrition, rhinitis, pain of rheumatic arthralgia	450332141114065LY	2	0.008
186	*Urceola huaitingii* (Chun & Tsiang) D. J. Middleton	Apocynaceae	Mao dong zhong teng	Hong dong zhong, Jiu niu teng	Woody vine	Root, Bark	Rheumatic bone pain, psychosis, dysmenorrhea, burn and scald	450332141116002LY	8	0.032
187	*Adina pilulifera* (Lam.) Franch. ex Drake	Rubiaceae	Shui tuan hua	Shui yang mei	Shrub	Root	Toothache, epistaxis, bruise, hyperostosis	450332150614027LY	4	0.016
188	*Damnacanthus giganteus* (Makino) Nakai	Rubiaceae	Duan ci hu ci	Chuan lian zhu, Ji jin shen	Shrub	Root	Hyperostosis, coccydynia	6–5177	1	0.004
189	*Damnacanthus indicus* C. F. Gaertn	Rubiaceae	Hu ci	Xiu hua zhen	Shrub	Whole plant	Liver cirrhosis, hepatitis	6–5079	3	0.012
190	*Gardenia jasminoides* J. Ellis	Rubiaceae	Zhizi	Shan zhi zi	Shrub	Fruit	Neonatal hepatitis, foot pain	450332150331056LY	3	0.012
191	*Hedyotis angustifolia* Miq	Rubiaceae	Xian hua er cao	Xia zi cao	Herb	Whole plant	Bruise	450332150614053LY	1	0.004
192	*Hedyotis caudatifolia* Merr. & F. P. Metcalf	Rubiaceae	Jian ye er cao	Guan yin cha	Herb	Whole plant	Amenorrhea, fallopian tube obstruction	450332150331029LY	2	0.008
193	*Hedyotis diffusa* Willd	Rubiaceae	Bai hua she she cao	She li cao	Herb	Whole plant	Hepatitis, nephritis, sweat stain	450332151021013LY	2	0.008
194	*Hedyotis hedyotidea* (DC.) Merr	Rubiaceae	Niu bai teng	Ji chang feng	Shrub	Root	Enteritis	450332141115040LY	2	0.008
195	*Mussaenda pubescens* W. T. Aiton	Rubiaceae	Yu ye jin hua	Bai zhi shan	Shrub	Whole plant	Sphagitis, furuncle, cold	450332141114017LY	1	0.004
196	*Paederia foetida* L	Rubiaceae	Ji shi teng	Ji shi teng	Herbaceous vine	Whole plant	Vulnerary, skin diseases, infantile malnutrition, strengthening spleen, disperse accumulations	450332141114070LY	1	0.004
197	*Serissa serissoides* (DC.) Druce	Rubiaceae	Bai ma gu	Liu yue xue	Shrub	Root	Infertility, infantile tracheitis	240	2	0.008
198	*Uncaria rhynchophylla* (Miq.) Miq. ex Havil	Rubiaceae	Gou teng	Ying zhua feng	Woody vine	Stem	Hyperostosis, lumbocrural pain, rheumatic bone pain, gout, high fever, upper hyperactivity of liver yang, psychosis, typhoid, dysentery	450332150331009LY	10	0.040
199	*Lonicera hypoglauca* Miq	Caprifoliaceae	Gu xian ren dong	Jin yin hua	Woody vine	Flower	Furuncle, amenorrhea, gynecological inflammation, cough, enteritis	450332171020003LY	4	0.016
200	*Artemisia anomala* S. Moore	Asteraceae	Qi hao	Bai hua cao	Herb	Whole plant	Dysmenorrhea, typhoid	450332151014022LY	2	0.008
201	*Artemisia argyi* H. Lév. & Vaniot	Asteraceae	Ai	Duan wu ai	Herb	Whole plant	Rheumatic bone pain, infertility, gynecological disorders	450332141114046LY	2	0.008
202	*Artemisia annua* L	Asteraceae	Huang hua hao	Qing hao	Herb	Whole plant	Cold, typhoid, hepatitis	450332150615031LY	2	0.008
203	*Aster scaber* Thunb	Asteraceae	Dong feng cai	Zuan shan gou	Herb	Root	Anesthetic, snake bite, bruise, undefined swelling and soreness	YY1602	1	0.004
204	*Bidens pilosa* L	Asteraceae	Gui zhen cao	Lao po cha	Herb	Whole plant	Cold, high fever, typhoid	450332141117006LY	2	0.008
205	*Carpesium abrotanoides* L	Asteraceae	Tian ming jing	Ye yan ye	Herb	Whole plant	Typhoid, psychosis	6–5036	1	0.004
206	*Centipeda minima* (L.) A. Braun & Asch	Asteraceae	Shi hu sui	E bu shi cao	Herb	Whole plant	Infantile abdominal distention, high fever, cold, infantile malnutrition, rhinitis, bruise, snake bite	450332150331048LY	2	0.008
207	*Chrysanthemum indicum* L	Asteraceae	Ye ju	Ye ju hua	Herb	Whole plant	Burn and scald, snake bite, conjunctivitis, furuncle, nephritis edema	450332141114047LY	5	0.020
208	*Cirsium japonicum* DC	Asteraceae	Da ji	Shan luo bo, Lei gong ci	Herb	Whole plant	Breast cancer, liver cancer, bruise	450332160512013LY	1	0.004
209	*Eclipta prostrata* (L.) L	Asteraceae	Li chang	Han lian cao, Mo han lian	Herb	Whole plant	Long menstrual period, dysentery, epistaxis, enteritis, furuncle	450332150821016LY	8	0.032
210	*Elephantopus scaber* L	Asteraceae	Di dan cao	Tu gong ying	Herb	Whole plant	Hemorrhoids, knife wound, toothache, diabetes, stomachache	450332141115033LY	2	0.008
211	*Emilia sonchifolia* (L.) DC	Asteraceae	Yi dian hong	Yi dian hong	Herb	Whole plant	Amenorrhea, posttraumatic ulcer, inguinal lymphadenopathy, hepatitis, improving eyesight, pneumonia	450332150614045LY	6	0.024
212	*Eupatorium fortunei* Turcz	Asteraceae	Pei lan	Ze lan	Herb	Whole plant	Set a broken bone	YY1603	2	0.008
213	*Gynura japonica* (Thunb.) Juel	Asteraceae	Ju san qi	Hong feng cai	Herb	Whole plant	Stop bleeding, promote tissue regeneration, metrorrhagia, set a broken bone	450332151020003LY	1	0.004
214	*Inula japonica* Thunb	Asteraceae	Xuan fu hua		Herb	Whole plant	Eye inflammation	YY1604	1	0.004
215	*Kalimeris indica* (L.) Sch. Bip	Asteraceae	Ma lan	Lu bian ju	Herb	Whole plant	Furuncle, typhoid	450332141114032LY	1	0.004
216	*Laggera alata* (D. Don) Sch.-Bip. ex Oliv	Asteraceae	Liu leng ju	Lu er ling	Herb	Whole plant	Stimulate the menstrual flow, rheumatism	450332150620006LY	1	0.004
217	*Ligularia japonica* (Thunb.) Less	Asteraceae	Da tou tuo wu	Du lian, Wu zhua qi, Nan gua qi	Herb	Root	Traumatic injury, lumbocrural pain, undefined swelling and soreness	YY1605	1	0.004
218	*Senecio scandens* Buch.-Ham. ex D. Don	Asteraceae	Qian li guang	Jiu li guang	Herb	Whole plant	Psoriasis, furuncle, insecticide, relieve itching	450332141114025LY	3	0.012
219	*Siegesbeckia orientalis* L	Asteraceae	Xi xian	Huang hua cao	Herb	Whole plant	Sciatica	YY1606	2	0.008
220	*Solidago decurrens* Lour	Asteraceae	Yi zhi huang hua	She tou wang	Herb	Whole plant	Snake bite, typhoid	450332151014021LY	1	0.004
221	*Acmella paniculata* (Wallich ex Candolle) R. K. Jansen	Asteraceae	Jin niu kou	Long zhu cao	Herb	Whole plant	Stomach ache and acid regurgitation, toothache	450332151019003LY	1	0.004
222	*Vernonia patula* (Aiton) Merr	Asteraceae	Xian xia hua	Gou zai hua	Herb	Whole plant	Typhoid, diarrhea	450332141114081LY	4	0.016
223	*Xanthium strumarium* L	Asteraceae	Cang er	Nian shen zi	Herb	Whole plant	Rhinitis, typhoid, prostatitis	381	4	0.016
224	*Youngia japonica* (L.) DC	Asteraceae	Huang an cai	Huang gua xiang	Herb	Whole plant	Yellow fluid ulcers, stone, furuncle	450332141115128LY	4	0.016
225	*Canscora lucidissima* (H. Lév. & Vaniot) Hand.-Mazz	Gentianaceae	Chuan xin cao	Chuan xian cao, Shi zi qian	Herb	Whole plant	Stomachache, hepatitis	YY1607	1	0.004
226	*Ardisia crenata* Sims	Primulaceae	Zhu sha gen	Tie liang san	Shrub	Root	Set a broken bone, bruise, costalgia, scabies	450332141114015LY	8	0.032
227	*Ardisia gigantifolia* Stapf	Primulaceae	Zou ma tai	Xue feng	Shrub	Root	Gout, gynecological inflammation, palsy, rheumatism	YY1608	1	0.004
228	*Ardisia japonica* (Thunb.) Blume	Primulaceae	Zi jin niu	Bu chu lin, Ai po cha	Shrub	Whole plant	Cough, pharyngitis	450332141116065LY	2	0.008
229	*Ardisia mamillata* Hance	Primulaceae	Hu she hong	Hong mao zhan	Shrub	Whole plant	Rheumatic bone pain, stop bleeding	450332141116052LY	1	0.004
230	*Lysimachia christiniae* Hance	Primulaceae	Guo lu huang	Guo ling long	Herb	Whole plant	Stone	450332150620002LY	1	0.004
231	*Lysimachia congestiflora* Hemsl	Primulaceae	Lin shi jiu	Guo lu huang	Herb	Whole plant	Bruise, analgesia, cholagogic	450332160515014LY	1	0.004
232	*Plumbago zeylanica* L	Plumbaginaceae	Bai hua dan	Po gu dan, Meng lao hu	Herb	Leaf	Infantile malnutrition	450332141115014LY	2	0.008
233	*Plantago asiatica* L	Plantaginaceae	Che qian	Fan shao cao, Ma guai cao	Herb	Whole plant	Stone, electric ophthalmia, bruise, furuncle, diarrhea, dysentery, nephritis edema, typhoid	450332150331018LY	13	0.052
234	*Campanumoea javanica* Blume	Campanulaceae	Jin qian bao	Tu dang shen	Herbaceous vine	Root	Leukemia, alopecia, woman lack of milk after childbirth, fatigue	450332150821021LY	1	0.004
235	*Codonopsis lanceolata* (Siebold & Zucc.) Trautv	Campanulaceae	Yang ru	Shan hai luo	Herbaceous vine	Root tuber	Woman lack of milk after childbirth	450332150821013LY	1	0.004
236	*Lobelia chinensis* Lour	Campanulaceae	Ban bian lian	Ban bian lian	Herb	Whole plant	Snake bite, stone, set a broken bone	450332150615011LY	3	0.012
237	*Lobelia angulata* G. Forst	Campanulaceae	Tong chui yu dai cao	Fu ming cao, Di yang mei	Herb	Whole plant	Eye disease, unclear vision	450332141115070LY	1	0.004
238	*Platycodon grandiflorus* (Jacq.) A. DC	Campanulaceae	Jie geng	Yang jie	Herb	Root	Rheumatic bone pain, cough	6–5312	2	0.008
239	*Lycianthes biflora* (Lour.) Bitter	Solanaceae	Hong si xian	Shi eqie	Herb	Whole plant	Snake bite, hypertension	450332141115043LY	2	0.008
240	*Lycium chinense* Mill	Solanaceae	GouQi	Di gu pi	Shrub	Root-bark	Hyperostosis, fallopian tube obstruction, gray hair	450332141115027LY	4	0.016
241	*Physalis angulata* L	Solanaceae	Ku zhi	Deng long pao	Herb	Whole plant	Sphagitis, typhoid, enteroptosis	450332141115111LY	3	0.012
242	*Solanum lyratum* Thunb	Solanaceae	Bai ying	Mao xiu cai	Herbaceous vine	Whole plant	Bad urination and defecation, cold, high fever, gastric perforation, inflammation, bleeding wound, rheumatic bone pain	450332141114010LY	6	0.024
243	*Cuscuta australis* R. Br	Convolvulaceae	Nan fang tusi zi	Wu gen teng	Herb	Whole plant	Stone, infantile malnutrition	450332150617013LY	1	0.004
244	*Dichondra repens* Forst	Convolvulaceae	Ma ti jin	Xiao guai zi yao, Luo di jin qian	Herb	Whole plant	Enteritis, hepatitis, gastric hemorrhage, stone, cholecystitis, oral ulcer, infantile malnutrition, traumatic injury	YY1609	1	0.004
245	*Buddleja asiatica* Lour	Scrophulariaceae	Bai bei feng	Si fang geng	Shrub	Stem and leaf	Typhoid	450332141118007LY	1	0.004
246	*Buddleja lindleyana* Fortune	Scrophulariaceae	Zui yu cao	Yang jiao pao	Shrub	Stem and leaf	Toothache, typhoid, insecticide, summer damp stomachache, abnormal leukorrhea, hemorrhoids	450332150617014LY	1	0.004
247	*Paulownia fortunei* (Seem.) Hemsl	Paulowniaceae	Bai hua pao tong	Pao tong xu	Tree	Root	Detumescence, analgesia	233	1	0.004
248	*Siphonostegia chinensis* Benth	Orobanchaceae	Yin xing cao	Tu yin chen	Herb	Whole plant	Neonatal jaundice	450332150618002LY	1	0.004
249	*Striga asiatica* (L.) Kuntze	Orobanchaceae	Du jiao jin	Dong jiao gan	Herb	Whole plant	Infantile malnutrition	6–5090	1	0.004
250	*Primulina eburnea* (Hance) Yin Z. Wang	Gesneriaceae	Niu er duo	Shi hu er	Herb	Rhizome	Dysentery, hyperostosis, gastritis, gastric perforation, metrorrhagia	450332141117064LY	3	0.012
251	*Primulina fimbrisepala* (Hand.-Mazz.) Yin Z. Wang	Gesneriaceae	Ma huang qi	Shi ma huang	Herb	Rhizome	Hyperostosis, rheumatism, bruise, enrich the blood, cooling blood, infantile malnutrition, bee sting, stomachache	450332150331013LY	5	0.020
252	*Campsis grandiflora* (Thunb.) K. Schum	Bignoniaceae	Ling xiao	Bai gou chang, Hong hua dao shui lian	Woody vine	Root	Rheumatic bone pain, traumatic injury, amenorrhea, hemorrhoids, enteritis	450332150618016LY	4	0.016
253	*Radermachera sinica* (Hance) Hemsl	Bignoniaceae	Cai dou shu	Niu wei shu	Tree	Root	Internal injury, liver cirrhosis, traumatic injury, lumbar disease	450332141117056LY	1	0.004
254	*Dicliptera chinensis* (L.) Juss	Acanthaceae	Gou gan cai	Gou gan cai	Herb	Whole plant	Infantile malnutrition	YY1610	1	0.004
255	*Justicia ventricosa* Wall. ex Hook. f	Acanthaceae	Hei ye xiao bo gu	Da bo gu	Shrub	Whole plant	Rheumatic bone pain	YY1611	1	0.004
256	*Strobilanthes cusia* (Nees) Kuntze	Acanthaceae	Ban lan	Ban lan gen, Ma lan	Shrub	Whole plant	Inguinal lymphadenopathy	YY1612	1	0.004
257	*Verbena officinalis* L	Verbenaceae	Ma bian cao	Shun ci cao	Herb	Whole plant	Stone, cirrhosis ascites	450332160511008LY	3	0.012
258	*Glechoma longituba* (Nakai) Kupr	Lamiaceae	Huo xue dan	Zuan di feng, Tou gu xiao	Herb	Whole plant	Stone, detumescence, analgesia, hyperostosis, eczema, mastitis	450332150330023LY	4	0.016
259	*Clerodendrum bungei* Steud	Lamiaceae	Chou mu dan	Chou mu dan	Shrub	Root	Arthralgia, hypertension, hemorrhoids, rectocele, sciatica, amenorrhea, gynecological disorders	YY1613	2	0.008
260	*Leonurus japonicus* Houtt	Lamiaceae	Yi mu cao	Yi mu cai	Herb	Whole plant	Fallopian tube obstruction, dysmenorrhea, infertility, amenorrhea	450332141115010LY	4	0.016
261	*Mentha canadensis* L	Lamiaceae	Bo he	Ye bao he	Herb	Whole plant	Neonatal cough, infantile high fever	450332141114045LY	3	0.012
262	*Perilla frutescens* (L.) Britton	Lamiaceae	Zi su	Zi su	Herb	Whole plant	Wind-heat type common cold, typhoid, measles syndrome	450332141114058LY	4	0.016
263	*Prunella vulgaris* L	Lamiaceae	Xia ku cao	Xia ku cao	Herb	Whole plant	Psychosis, conjunctivitis, tumor, herpes zoster, hepatitis	450332150413012LY	5	0.020
264	*Scutellaria barbata* D. Don	Lamiaceae	Ban zhi lian	Ya shua cao	Herb	Whole plant	Nasal polyps, snake bite, hepatitis B, hepatitis, knife wound	450332150401038LY	7	0.028
265	*Stachys geobombycis* C. Y. Wu	Lamiaceae	Di can	Bai chong cao	Herb	Whole plant	Tuberculosis, tracheitis, pneumonia	YY1614	1	0.004
266	*Sagittaria trifolia* L	Alismataceae	Ye ci gu	Jian dao cao	Herb	Root tuber	Furuncle, bruise, sphagitis	YY1615	1	0.004
267	*Eriocaulon buergerianum* Körn	Eriocaulaceae	Gu jing cao		Herb	Inflorescence	Stone	YY1616	1	0.004
268	*Alpinia sichuanensis* Z. Y. Zhu	Zingiberaceae	Jian gan feng	Jian gan feng	Herb	Rhizome	Heatstroke, rheumatic bone pain	450332141115125LY	3	0.012
269	*Curcuma aromatica* Salisb	Zingiberaceae	Yu jin	Mao jiang huang	Herb	Rhizome	Acute hepatitis, liver ascites	YY1617	1	0.004
270	*Curcuma longa* L	Zingiberaceae	Jiang huang	Huang jiang	Herb	Rhizome	Stone, cyst, mastopathy, bruise, rheumatic bone pain	YY1618	5	0.020
271	*Curcuma phaeocaulis* Valeton	Zingiberaceae	E zhu	Wu xin jiang, Hei xin jiang, Wu qi	Herb	Rhizome	Traumatic injury, hyperostosis, irregular menstruation	YY1619	2	0.008
272	*Zingiber officinale* Roscoe	Zingiberaceae	Jiang	Sheng jiang	Herb	Rhizome	Long menstrual period, infantile diarrhea, wind-heat type common cold, vomiting, high fever, vomiting of pregnancy, bald spot	YY1620	10	0.040
273	*Aletris spicata* (Thunb.) Franch	Nartheciaceae	Fen tiao er cai	Jin xian diao bai mi	Herb	Root	Syphilis	YY1621	1	0.004
274	*Asparagus cochinchinensis* (Lour.) Merr	Asparagaceae	Tian men dong	Tian dong	Herb	Root tuber	Scabies	450332150329006LY	1	0.004
275	*Hosta ventricosa* (Salisb.) Stearn	Asparagaceae	Zi e	Wan nian chun, Zi yu zan	Herb	Whole plant, Root	Undefined swelling and soreness, bone sticking throat, carbuncle	450332150617022LY	1	0.004
276	*Ophiopogon japonicus* (L. f.) Ker Gawl	Asparagaceae	Mai dong	Xi ye mai dong	Herb	Root tuber	The first bite of food for a newborn, constipation, cough, hepatitis	42858	4	0.016
277	*Polygonatum cyrtonema* Hua	Asparagaceae	Duo hua huang jing	Huang jing	Herb	Rhizome	Diabetes, infertility, hyperglycemia	450332150411039LY	2	0.008
278	*Reineckea carnea* (Andrews) Kunth	Asparagaceae	Ji xiang cao	Gan yan cao, Fei ke cao	Herb	Whole plant	Infantile malnutrition, hepatitis	YY1622	2	0.008
279	*Paris polyphylla* Sm	Melanthiaceae	Qi ye yi zhi hua	Zhong lou	Herb	Rhizome	Snake bite, hepatitis B, numbness of hands and feet, undefined swelling and soreness, lymphadenopathy	YY1623	2	0.008
280	*Smilax china* L	Smilacaceae	Ba qia	Jin gang dou	Shrub	Rhizome	Arthralgia, rheumatism	450332150819015LY	3	0.012
281	*Smilax glabra* Roxb	Smilacaceae	Tu fu ling	Tu fu ling	Shrub	Rhizome	Abnormal leukorrhea, eczema	450332151023004LY	1	0.004
282	*Smilax riparia* A. DC	Smilacaceae	Niu wei cai	Niu wei jie	Herbaceous vine	Root	Acute hepatitis, liver ascites	450332150412019LY	1	0.004
283	*Acorus calamus* L	Acoraceae	Chang pu	Shui changpu	Herb	Whole plant	Long menstrual period, moist heat	YY1624	2	0.008
284	*Acorus tatarinowii* Schott	Acoraceae	Shi chang pu	Shi chang pu	Herb	Root	Uterine cold stomachache, sciatica, tinnitus, rheumatic bone pain	450332160512008LY	2	0.008
285	*Alocasia cucullata* (Lour.) G. Don	Araceae	Jian wei yu	Lao hu yu	Herb	Root tuber	Bruise	YY1625	1	0.004
286	*Amydrium hainanense* (K. C. Ting & T. L. Wu ex H. Li, Y. Shiao & S. L. Tseng) H. Li	Araceae	Chuan xin teng	Chuan xin feng	Herbaceous vine	Stem and Leaf	Hepatitis, nephritis edema, gastritis, stomachache(stewing with pig's stomach)	450332150630001LY	2	0.008
287	*Arisaema erubescens* (Wall.) Schott	Araceae	Yi ba san nan xing	Tian nan xing	Herb	Tuber	Snake bite	450332151015052LY	1	0.004
288	*Pinellia ternata* (Thunb.) Breitenb	Araceae	Ban xia	Ban xia	Herb	Tuber	Headache, vomiting of pregnancy, hyperthyroidism	450332150821055LY	2	0.008
289	*Lycoris radiata* (L'Hér.) Herb	Amaryllidaceae	Shi suan	Hong hua shi suan	Herb	Bulb	Acute mastitis, undefined swelling and soreness	YY1626	1	0.004
290	*Belamcanda chinensis* (L.) Redouté	Iridaceae	She gan	Bian xu	Herb	Rhizome	Sore throat, hepatitis	383	1	0.004
291	*Iris japonica* Thunb	Iridaceae	Hu die hua	Zhan long jian, Tong qi	Herb	Rhizome	Hepatitis, rabies, bee sting, snake bite	YY1627	3	0.012
292	*Stemona tuberosa* Lour	Stemonaceae	Da bai bu	Bai bu	Herbaceous vine	Root tuber	Cough	450332150402027LY	1	0.004
293	*Dioscorea bulbifera* L	Dioscoreaceae	Huang du	Huang yao zi, Jin xian diao dan	Herbaceous vine	Root tuber	Breast cancer, liver cancer, hyperthyroidism	211	3	0.012
294	*Schizocapsa plantaginea* Hance	Dioscoreaceae	Lie guo shu	Shui tian qi	Herb	Root tuber	Sore throat, toothache, infantile pharyngitis, sprain swelling, lymphadenopathy	450332150821007LY	1	0.004
295	*Pandanus austrosinensis* T. L. Wu	Pandanaceae	Lu dou cao	Ye bo luo	Herb	Fruit	Long menstrual period, rheumatic bone pain, stone, gynecological cyst	450332150619038LY	2	0.008
296	*Curculigo orchioides* Gaertn	Hypoxidaceae	Xian mao	Du jiao huang mao	Herb	Rhizome	Male infertility, gynecological disorders, alopecia	450332150409007LY	1	0.004
297	*Nervilia fordii* (Hance) Schltr	Orchidaceae	Mao chun yu lan	Qing tian kui	Herb	Whole plant	Hepatitis B	YY1628	1	0.004
298	*Spiranthes sinensis* (Pers.) Ames	Orchidaceae	Shou cao	Zhan long cao, Pan long shen	Herb	Whole plant	Herpes zoster, nourishing yin benefiting qi, cooling blood, detoxification	450332150402032LY	1	0.004
299	*Juncus effusus* L	Juncaceae	Deng xin cao	Zuan di wu gong	Herb	Whole plant	Stone pain, herpes zoster, renal colic	450332150411045LY	4	0.016
300	*Cyperus rotundus* L	Cyperaceae	Xiang fu zi	Xiang tou cao	Herb	Root	Fallopian tube obstruction, enteritis, infertility	450332150614050LY	3	0.012
301	*Eleocharis dulcis* (Burm. f.) Trin. ex Hensch	Cyperaceae	BiQi	Ma ti	Herb	Corm	Eye pain	YY1629	1	0.004
302	*Phyllostachys nigra* (Lodd. ex Lindl.) Munro	Poaceae	Zi zhu	Hei zhu	Herb	Stem	Rheumatic bone pain, enteroptosis	YY1630	2	0.008
303	*Coix lacryma-jobi* L	Poaceae	YiYi	Lao ya cao	Herb	Root	Infertility, rheumatism, stone, bad urination and defecation, hemorrhoids, nephritis edema, moist heat	450332141118004LY	6	0.024
304	*Imperata cylindrica* var. *major* (Nees) C. E. Hubbard	Poaceae	Da bai mao	Bai mao gen	Herb	Rhizome	Epistaxis, typhoid, stroke	450332141117010LY	9	0.036
305	*Lophatherum gracile* Brongn	Poaceae	Dan zhu ye	Shan ji mi	Herb	Whole plant	Typhoid, cold, sphagitis	YY1631	1	0.004
306	*Phragmites karka* (Retz.) Trin. ex Steud	Poaceae	Ka kai lu	Lu gen, Guo jiang long	Herb	Rhizome	Stone, cold, typhoid, stomachache, hepatitis, eye inflammation	YY1632	6	0.024

**Table 5:** The diversity of medicinal plants used by local healers in Gongcheng

Family	Number of species	Percentage of species (%)
Asteraceae	25	8.17
Fabaceae	17	5.56
Rubiaceae	12	3.92
Rutaceae	9	2.94
Rosaceae	8	2.61
Lamiaceae	8	2.61
Malvaceae	8	2.61
Polygonaceae	8	2.61
Vitaceae	7	2.29
Primulaceae	6	1.96
Poaceae	5	1.63
Euphorbiaceae	5	1.63
Asparagaceae	5	1.63
Campanulaceae	5	1.63
Araliaceae	5	1.63
Zingiberaceae	5	1.63
Solanaceae	4	1.31
Polypodiaceae	4	1.31
Araceae	4	1.31
Apiaceae	4	1.31
Apocynaceae	4	1.31
Amaranthaceae	4	1.31
Moraceae	4	1.31
Aristolochiaceae	4	1.31
Schisandraceae	4	1.31
Aquifoliaceae	4	1.31
Ranunculaceae	3	0.98
Acanthaceae	3	0.98
Menispermaceae	3	0.98
Lauraceae	3	0.98
Smilacaceae	3	0.98
Others	113	37.01
Total	306	100.00

In general, the distribution of medicinal plant species in various families was relatively scattered, and the selection of medicinal plants by local healers was highly diverse, indicating that local healers were competent at using a variety of medicinal plants to treat various diseases. Hence, the mountains with ideal habitat and high biodiversity are called the "*Yao mountains*" (Fig. 1), and the Yao people have a traditional custom of collecting herbs from the "*Yao mountains*".

**Fig. 6:**
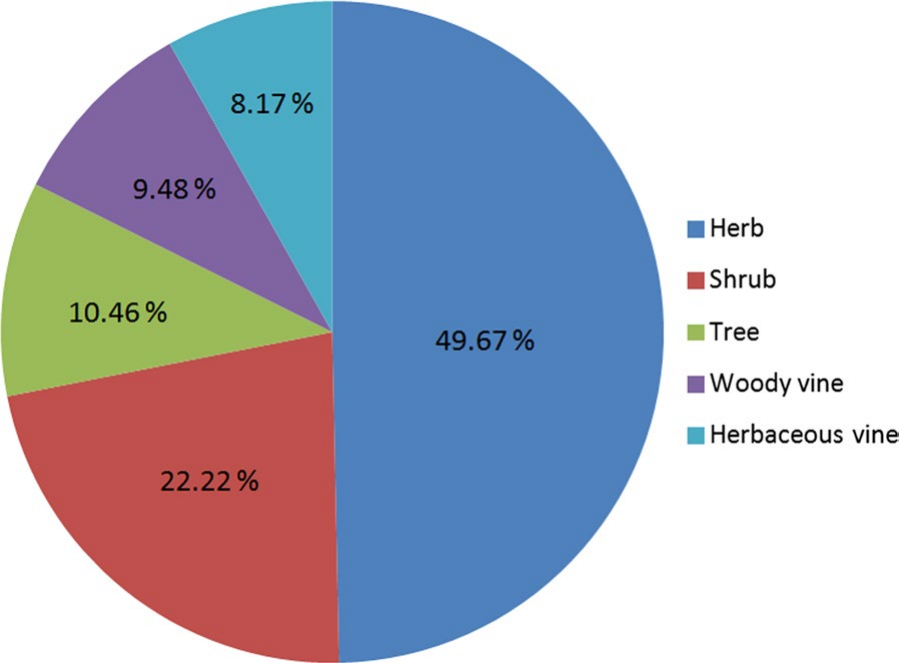
Life forms of medicinal plants in the study area

The medicinal plants observed in this study were classified into 152 species of herb (49.67%), 68 species of shrub (22.22%), 32 species of tree (10.46%), 29 species of the woody vine (9.48%), and 25 species of the herbaceous vine (8.17%) (Fig. 6). Herbs were most numerous and accounted for around half of the total species, because most herbs are easy to pick, cultivate and reproduce, and are convenient for use. These results are consistent with other research [44, 46–49]. In addition, the medicinal plants used by local healers fell into various life forms, which demonstrated that local healers had experimented with the use of an extensive range of plants over hundreds of years and had finally amassed the unique knowledge and experience of Yao medicine as we find it today.

The efficacy of medicinal plants is closely related to the medicinal part used. Different medicinal parts of the same plant may have different efficacy, and the same medicinal part may have different efficacy in different prescriptions. There were 330 medicinal parts belonging to 306 medicinal plants in Gongcheng, which were treated as 330 medicinal species. Among them, whole plants were the greatest in number (125 species), accounting for 37.88% of the total species, followed by roots (20.30%), rhizomes (7.27%), stems (7.27%), root tubers (4.55%), leaves (4.24%), stem and leaves (4.24%), barks (including root-barks) (3.94%), fruits (including legume and rinds) (3.64%), seeds (2.12%), flowers (including inflorescence) (1.82%), and others (including bulbs, bulbils, corms, tuber, stem pith, and thorns) (2.73%) (Table 6). Among the 330 medicinal parts used by the local healers in Gongcheng, there were two main categories of whole plants and roots, in a total of 192 species, that accounted for 62.75% of the total species. Similar results have been found in some minority communities of Guangxi [7, 8, 27, 36, 50]. The local healers generally believe that roots are where the plant's medicinal powers converge, and their efficacy is optimal. Among whole plants, most are herbs, because herbs are easy to pick, and their habitats are diverse.

**Table 6:** Medicinal plant parts used by local healers in Gongcheng

Medicinal parts	Species	Percentage (%)	Medicinal parts	Species	Percentage (%)
Whole plants	125	37.88	Stem and leaves	14	4.24
Roots	67	20.30	Barks	13	3.94
Rhizomes	24	7.27	Fruits	12	3.64
Stems	24	7.27	Seeds	7	2.12
Root tuber	15	4.55	Flowers	6	1.82
Leaves	14	4.24	Others	9	2.73

### Relative frequency of citation

The RFC evaluates important plant species used by local healers to treat various diseases. From the 248 prescriptions investigated, the number of prescriptions mentioning plant species (FC) used ranged from one to 14. Calculations showed that 33 medicinal plant species had an FC > 5 (Table 7). The RFC value calculated for these 33 medicinal plant species ranged from 0.024 to 0.056. The higher RFC values included *Kadsura longipedunculata*, *Schefflera heptaphylla*, and *Plantago asiatica*.

**Table 7:** Relative frequency of citation (RFC) of plant species mentioned in prescriptions

Scientific name	FC	RFC	Scientific name	FC	RFC
*Kadsura longipedunculata*	14	0.056	*Equisetum ramosissimum*	7	0.028
*Schefflera heptaphylla*	14	0.056	*Drynaria roosii*	7	0.028
*Plantago asiatica*	13	0.052	*Clematis chinensis*	7	0.028
*Achyranthes aspera*	12	0.048	*Senna tora*	7	0.028
*Sanguisorba officinalis*	12	0.048	*Ventilago leiocarpa*	7	0.028
*Flemingia prostrata*	12	0.048	*Sabia japonica*	7	0.028
*Aconitum carmichaeli*	10	0.040	*Eleutherococcus nodiflorus*	7	0.028
*Tetrastigma hemsleyanum*	10	0.040	*Cynanchum paniculatum*	7	0.028
*Uncaria rhynchophylla*	10	0.040	*Scutellaria barbata*	7	0.028
*Zingiber officinale*	10	0.040	*Urena lobata*	6	0.024
*Bupleurum marginatum*	9	0.036	*Semiliquidambar cathayensis*	6	0.024
*Imperata cylindrica* var. *major*	9	0.036	*Eucommia ulmoides*	6	0.024
*Akebia trifoliata*	8	0.032	*Emilia sonchifolia*	6	0.024
*Polygala fallax*	8	0.032	*Solanum lyratum*	6	0.024
*Urceola huaitingii*	8	0.032	*Coix lacryma-jobi*	6	0.024
*Eclipta prostrata*	8	0.032	*Phragmites karka*	6	0.024
*Ardisia crenata*	8	0.032			

The higher the RFC value, the more familiar was the local healers with the species; furthermore, and of great importance, the species were abundant and easy to obtain locally. Ten of these 33 medicinal plant species were traditional Laoban medicines, indicating that the local healers were good at using traditional Laoban medicines to treat diseases, especially *Kadsura longipedunculata* (the Laoban medicine name is *xiao zuan*) in the treatment of rheumatism. It also showed that local healers had a long history of using Laoban medicines, including *Achyranthes aspera* (the Laoban medicine name is *niu xi feng*) for the treatment of hyperostosis and rheumatic bone pain, *Uncaria rhynchophylla* (the Laoban medicine name is *ying zhua feng*) treatment for hyperostosis, lumbocrural pain, rheumatic bone pain, and others [26, 27]. These were all traditional and common usages in the local area.

### Protect Yao traditional medicinal knowledge and medicinal plants

As for the protection of Gongcheng Yao traditional medicinal knowledge, the local government should provide a better environment for Yao healers, consider the legality of medical practice for Yao healers and give appropriate advertisements for those Yao healers. The local government also may pay more attention to the inheritance of Yao traditional medicinal knowledge and set up training course for young people. We firmly believe that the training of young personnel will strongly support the sustainable development of Yao medicine [35] and also is a very important approach for the conservation of Yao traditional medicinal knowledge.

Based on the demographic investigation, the Yao healers in Gongcheng aged over 60 more than half, some Yao healers are dying out, but their traditional medicinal knowledge was not be documented, so the further survey and record of Yao traditional medicinal knowledge is imperative [51], especially Sanjiang and Guanying townships in Gongcheng. Books and scientific reports about medicinal plants and Yao traditional medicinal knowledge should be published [8, 52].

In order to enhance the public understanding and confidence, as well as the safety of Yao traditional medicines, the advanced theories and methods of pharmacology, phytochemistry, and molecular pharmacognosy should be applied to study the Yao traditional medicines and traditional medicinal knowledge [8, 52]. And also in order to conserve local medicinal plant resources, the local government should encourage Yao people to plant preferred or rare medicinal plants in their farmlands [8, 35, 51, 52], which also in line with the strategy of rural revitalization.

## Conclusion

In this study, we analyzed the data collected from 352 local healers in nine townships of Gongcheng, the Guanyin and Sanjiang townships had the highest distribution of per capita healers, so these two townships were key areas for the protection inheritance of traditional medicinal knowledge. Our investigation recorded 306 medicinal plant species (belonging to 116 families and 255 genera). Most local healers are good at treating traumatic injury and orthopedics, digestive system, skin disease and rheumatic disease. Herbal plants were most commonly used among the medicinal plant species, with whole plants and roots being favored. The most commonly used medicinal method was decoction, and the use of plasters, creams, and some form of moxibustion and cupping skills also showed local practice.

The demographics of local healers in Gongcheng demonstrate a decreasing number of local healers, aging of healers, lack of successors, and the loss of Yao traditional medicinal knowledge. These are affected by modern medicine, urbanization and economic development, and the conservative manner and oral mode of transmitting medicinal knowledge to the next generation. The Yao people excel at using rich medicinal plants to treat various diseases in Gongcheng, which reflects their profound wisdom. The local healers' rich knowledge of traditional medicine and unique remedies make the treatments convenient and efficient, and they have strong regional characteristics. Based on the profound local Yao medicinal knowledge, Gongcheng is currently building the Panwang Medicinal Valley, Yao-Han Health Center and Yao Medical Hospital, for which the current study also provides preliminary data and guidelines. The inheritance of Yao traditional medicinal knowledge is inseparable from the rich medicinal plant resources in the "*Yao mountains*". Therefore, while attempting to rescue the local traditional medicinal knowledge, great attention should also be paid to biodiversity conservation. Only by addressing both these factors can traditional medicinal knowledge be effectively inherited and developed.

## Data Availability

All data generated or analyzed during this study are included in this published article.
